# Mitochondria-targeting Cu_3_VS_4_ nanostructure with high copper ionic mobility for photothermoelectric therapy

**DOI:** 10.1126/sciadv.adi9980

**Published:** 2023-11-01

**Authors:** Yushan Dong, Shuming Dong, Chenghao Yu, Jing Liu, Shili Gai, Ying Xie, Zhiyu Zhao, Xiran Qin, Lili Feng, Piaoping Yang, Yanli Zhao

**Affiliations:** ^1^ Key Laboratory of Superlight Materials and Surface Technology, Ministry of Education, College of Material Science and Chemical Engineering, Harbin Engineering University, Harbin 150001, P. R. China.; ^2^ Key Laboratory of Functional Inorganic Material Chemistry, Ministry of Education, School of Chemistry and Materials Science, Heilongjiang University, Harbin, 150080, P. R. China.; ^3^ Department of Ultrasound, The First Affiliated Hospital of Harbin Medical University, Harbin 150001, P. R. China.; ^4^ School of Chemistry, Chemical Engineering and Biotechnology, Nanyang Technological University, 21 Nanyang Link, Singapore 637371, Singapore.

## Abstract

Thermoelectric therapy has emerged as a promising treatment strategy for oncology, but it is still limited by the low thermoelectric catalytic efficiency at human body temperature and the inevitable tumor thermotolerance. We present a photothermoelectric therapy (PTET) strategy based on triphenylphosphine-functionalized Cu_3_VS_4_ nanoparticles (CVS NPs) with high copper ionic mobility at room temperature. Under near-infrared laser irradiation, CVS NPs not only generate hyperthermia to ablate tumor cells but also catalytically yield superoxide radicals and induce endogenous NADH oxidation through the Seebeck effect. Notably, CVS NPs can accumulate inside mitochondria and deplete NADH, reducing ATP synthesis by competitively inhibiting the function of complex I, thereby down-regulating the expression of heat shock proteins to relieve tumor thermotolerance. Both in vitro and in vivo results show notable tumor suppression efficacy, indicating that the concept of integrating PTET and mitochondrial metabolism modulation is highly feasible and offers a translational promise for realizing precise and efficient cancer treatment.

## Teaser

Triphenylphosphine-modified Cu_3_VS_4_ nanoparticles with copper ionic mobility are developed for photothermoelectric tumor therapy.

## INTRODUCTION

Photothermal therapy (PTT), which transforms near-infrared (NIR) light energy into thermal energy by using photothermal agents, has been considered as a potential clinical and preclinical strategy for cancer treatment in recent decades because of its noninvasiveness, high spatiotemporal precision, and high spatial specificity (*1*–*4*). However, cancer cells are inevitably stimulated by hyperthermia in bodies, and thus, the expression of heat shock proteins (HSPs) is boosted, which endows cells with undesired heat tolerance and thus substantially compromises the efficacy of PTT (*5*–*7*). Traditionally, limited by their acute cytotoxicity, poor tumor-targeting effect, and asynchronous hysteretic therapeutic effects, HSP inhibitors are generally incapable of silencing HSPs in complex physiological conditions securely and efficiently (*8*–*10*). Therefore, it is imperative to provide an efficient treatment strategy to suppress the expression of HSPs and enhance the efficacy of PTT. Proverbially, relying on the energy supplied by the adenosine triphosphate (ATP), the expression of HSPs (especially HSP70 and HSP90) can be down-regulated by inhibiting ATP synthesis to reverse the heat tolerance of tumors in PTT (*11*–*13*). Admittedly, glycolysis metabolism as the classical “Warburg effect” is traditionally considered as the primary energy source in cancer cells, but oxidative phosphorylation (OXPHOS) still serves as the main energy production pathway for a sizeable proportion of cells (*14*–*17*). During the OXPHOS metabolism, ATP is generated by transferring electrons to a series of large multisubunit protein assemblies (complexes I to IV) on the inner mitochondrial membrane. Specifically, complex I {NADH [reduced form of nicotinamide adenine dinucleotide (NAD^+^)] dehydrogenase} plays an essential role throughout the OXPHOS process by catalyzing NADH oxidation and functioning as the entry point of electrons into respiratory chain complexes (*18*, *19*). To sum up, the function of complex I as an appealing target can be competitively inhibited by consuming intracellular NADH, substantially suppressing ATP synthesis, and reducing HSPs expression. Moreover, PTT has limited efficacy as a monotherapy, making it necessary to combine PTT with other treatment options.

Actually, heat acts solely as a kind of output energy to ablate cancer cells throughout the PTT process. Inspired by the photothermoelectric (PTE) effect based on light-thermal-electricity energy conversion, heat can also be used as an energy input, and thermoelectric materials can be used for converting thermal energy into electrical energy and promoting the generation of reactive oxygen species (ROS) to induce tumor cells apoptosis (*20*–*23*). In this paper, we developed PTE therapy (PTET), which greatly eliminates the shortcomings of PTT. More specifically, the PTET is achieved on the basis of the photothermal and thermoelectric conversion processes. When a kind of PTE material is illuminated by light, a local temperature will rise under the photothermal effect, and the temperature gradient within the material drives the diffusion of charge carriers from the hot end to the cold end, thereby building up an electric potential difference; this process is known as the Seebeck effect (*24*–*27*). Recently, active materials with remarkable PTE properties, including graphene, MoS_2_, and Bi_2_Te_3_, have been extensively studied (*28*–*32*). Notably, low-cost copper chalcogenides become highly potential PTE materials because of their proper bandgaps and phonon-liquid characteristics. Classical copper chalcogenides, such as Cu_2_E or Cu_2−*x*_E (E = S/Se), have highly complex crystal structures at low temperatures and undergo phase transitions from the noncubic to cubic structure with increasing temperature (*33*–*35*). In the high-temperature phase, the E atoms maintain a rigid crystalline sublattice, whereas the Cu ions are disorderly distributed throughout many possible positions with liquid-like mobility. This “phonon-liquid-electron-crystal” model allows these materials to conduct phonons like liquids and electrons like crystals, bringing out many new and unusual electrical and thermal transport properties, containing ultralow thermal conductivity, high electrical conductivity, and high thermoelectric figure of merit ZT (*36*–*39*). Nevertheless, the high thermoelectric performance of Cu_2_E or Cu_2−*x*_E is only achieved at extremely high temperatures (800 to 1000 K) (*40*–*42*). It is believed that PTET agents should operate well near body temperature for in vivo application. Thus, copper chalcogenides are challenged in PTET applications regarding how to obtain a relatively pure cubic phase at room temperature for thermoelectric performance.

**Fig. 1.**
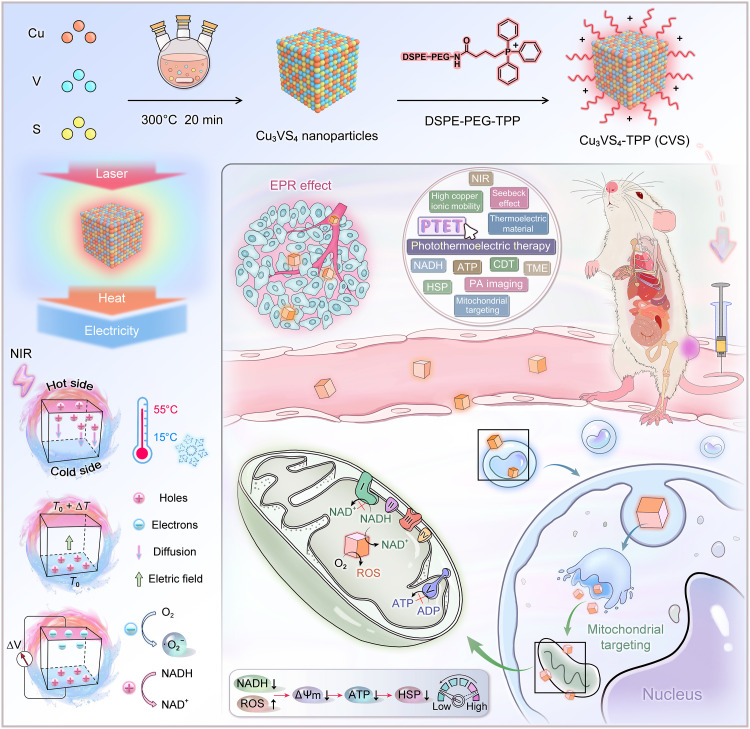
Scheme of the synthetic procedure and synergistic cancer PTET of CVS NPs. CVS NPs can convert NIR light into heat, creating a temperature difference between the two ends of the nanostructure. The electron-hole pairs effectively participate in redox reactions on the nanostructure surface, where electrons reduce O_2_ molecules to produce **·**O_2_^−^ and holes oxidize NADH through the Seebeck effect. Depletion of NADH by CVS NPs competitively inhibits the activity of mitochondrial respiratory complex I, thereby suppressing the ATP supply and down-regulating the expression of HSPs. Last, the undesired heat tolerance of tumor cells is alleviated during PTET. This domino effect facilitates the cell to approach apoptosis.

In this work, the ternary copper-based chalcogenide compound Cu_3_VS_4_ nanoparticles (NPs) with cubic structure (space group P$\bar{4}$3m) at room temperature were synthesized and investigated. Density functional theory (DFT) calculations have been performed to predict the threefold degeneracy occurring at the top of the valence band (VB), showing a promoting effect on thermoelectric performance. In particular, nudged elastic band (NEB) calculations revealed a relatively low diffusion barrier of Cu ions within the lattice at room temperature, thus demonstrating that the three-dimensional (3D) channel system in Cu_3_VS_4_ crystal structure allows interstitial Cu ions to migrate freely like a liquid. Cu_3_VS_4_ has been measured to exhibit an ultralow thermal conductivity of 0.3 W m^−1^ K^−1^ at room temperature. In addition, the introduction of V ions renders Cu_3_VS_4_ an intermediate bandgap (IB) semiconductor where the transient optical response would cause nonradiative relaxation of carriers excited from the VB to the IB, followed by ultrafast thermalization of the NP lattice. Together, the p-type ternary compound Cu_3_VS_4_ not only efficiently converts NIR light into heat but also has favorable thermoelectric performance at room temperature, showing great potential as a novel PTE material for PTET. Furthermore, Cu_3_VS_4_ NPs were modified by using distearoyl phosphoethanolamine–(polyethylene glycol)–triphenylphosphonium bromide (DSPE-PEG-TPP) to form Cu_3_VS_4_-TPP NPs (CVS NPs) with the mitochondrial-targeting ability for photoacoustic (PA) imaging–guided synergistic PTET/chemodynamic therapy (CDT) (Fig. 1). Under 808-nm laser irradiation, the produced CVS NPs exhibit prominent photothermal conversion efficiency (32.78%). Meanwhile, the finite difference time domain (FDTD) simulation confirmed the electric field enhancement near the NPs and the temperature difference between the two ends. Noticeably, the electron-hole pairs excited at the temperature gradient could be effectively separated to participate in redox reactions on the NP surface; specifically, electrons (e^−^) play a role in the reduction of oxygen (O_2_) molecules to produce superoxide radicals (**·**O_2_^−^), while holes (h^+^) are involved in NADH oxidation. CVS NPs competitively inhibit the activity of mitochondrial respiratory complex I by depleting NADH, thereby suppressing the ATP supply and down-regulating the expression of HSPs. Last, the undesired heat tolerance of tumor cells is alleviated during PTET. This “domino effect” facilitates the cell to approach apoptosis. Moreover, in the mild acidic tumor microenvironment (TME), CVS NPs are capable of catalyzing hydrogen peroxide (H_2_O_2_) to yield toxic hydroxyl radicals (**·**OH) for CDT through a Fenton-like reaction. In both in vitro and in vivo measurements, we control the temperature gradient (15° to 55°C) by an external cold source and 808-nm laser excitation to maximize the temperature gradient, thus greatly potentiating the thermoelectric effect. These prominent PTE/chemodynamic features endow the CVS NPs with effective antitumor performance and negligible biosafety concerns, demonstrating the proof-of-concept application of “phonon-liquid” PTE materials for cancer therapy.

## RESULTS

### Preparation and characterization of CVS NPs

**Fig. 2.**
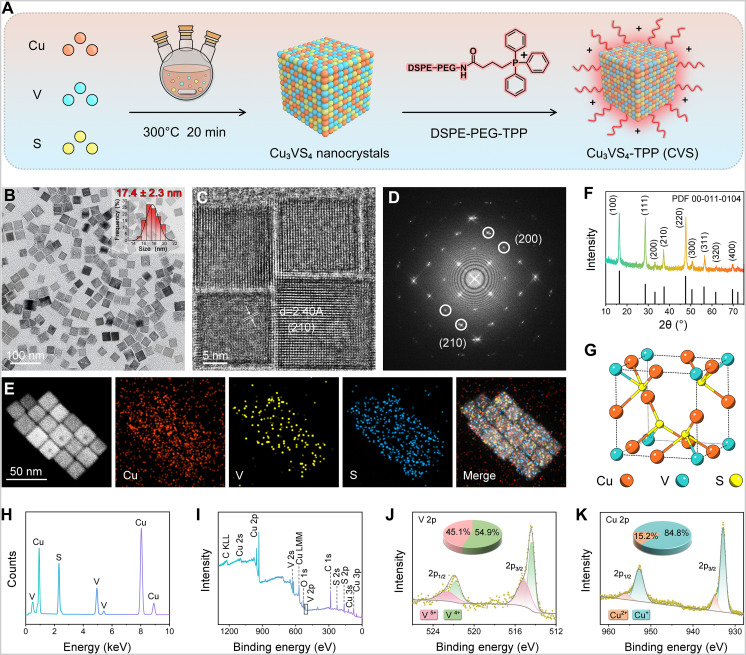
Preparation and characterizations. (**A**) Schematic illustration of the synthetic procedure of CVS NPs. (**B**) Low-magnification TEM images of Cu_3_VS_4_ NPs. The inset indicates the size distribution histogram determined by TEM. (**C**) High-resolution TEM image and (**D**) the corresponding fast Fourier transform patterns of Cu_3_VS_4_ NPs. (**E**) High-angle annular dark-field imaging–scanning TEM and the elemental mapping images of Cu_3_VS_4_ NPs. (**F**) PXRD patterns, (**G**) crystal structure, (**H**) EDS survey spectrum, and (**I**) survey XPS spectrum of Cu_3_VS_4_ NPs. (**J**) V 2p and (**K**) Cu 2p core-level XPS spectra.

The synthesis procedure of CVS NPs was illustrated in Fig. 2A. First, Cu_3_VS_4_ NPs were prepared according to a modified method, in which the sulfur precursor solution was mixed with the metal precursor solution via a thermal injection strategy (*43*, *44*). The transmission electron microscopy (TEM) image shows that the Cu_3_VS_4_ NPs have a uniform cubic shape with an average diameter of 17.4 nm (Fig. 2B). The high-resolution TEM image exhibits that the interplanar lattice spacing of ~0.24 nm was identified as (210) crystal plane of the cubic phase (Fig. 2C). The corresponding selected area electron diffraction (SEAD) pattern clearly exhibits the single-crystal nature, and the diffraction points can be indexed to the (200) and (210) planes of cubic Cu_3_VS_4_ (Fig. 2D). Elemental mapping analysis (Fig. 2E) highlights the homogeneous element distribution throughout the Cu_3_VS_4_ NPs, demonstrating the successful preparation. The powder x-ray diffraction (PXRD) pattern of Cu_3_VS_4_ NPs is presented in Fig. 2F, whose diffraction peaks can be matched well with the pure cubic Cu_3_VS_4_ phase (PDF 00-011-0104) with the lattice parameter of a = 5.393 Å (Fig. 2F). The high-symmetry cubic crystal structure belongs to the space group P$\bar{4}$3m (Fig. 2G), in which the corners of the cell are occupied by V ions, while the cube edges are occupied by Cu ions, and each metal ion is tetrahedrally coordinated with four S ions. Because the center of the cube is empty, the tetrahedral sites formed by the anions form a 3D channel system in the lattice contributing to the high ion mobility of Cu interstitial ions (*45*). The coexistence of Cu, V, and S elements was demonstrated through the energy-dispersive x-ray spectroscopy (EDS) spectrum (Fig. 2H). Comprehensive insights into the surface composition and valence state information of Cu_3_VS_4_ NPs were obtained through the utilization of x-ray photoelectron spectroscopy (XPS). The as-synthesized Cu_3_VS_4_ NPs mainly consisted of Cu, V, and S elements (Fig. 2I).

The high-resolution V 2p XPS spectrum of Cu_3_VS_4_ NPs can be assigned to the spin-splitting orbits of 2p_1/2_ and 2p_3/2_, and the area ratio between the two energy levels was fixed to a 1:2 ratio with an energy separation of 7.45 eV (Fig. 2J). This result confirms the coexistence of V^4+^ (54.9%) and V^5+^ (45.1%) in Cu_3_VS_4_ NPs. Meanwhile, two peaks can be resolved in the Cu 2p XPS spectrum at the binding energies of 952.6 and 932.8 eV with the spin-orbit splitting of 19.8 eV, which were attributed to Cu 2p_1/2_ and Cu 2p_3/2_ states, severally (Fig. 2K). The proportion of Cu^+^ and Cu^2+^ are estimated to be 84.8 and 15.2% by the ratio of integrated peak areas, respectively. The XPS spectrum of the S 2p region is also given in fig. S1. To endow the Cu_3_VS_4_ NPs with the mitochondria-targeting ability and higher biocompatibility, which were further functionalized with DSPE-PEG-TPP to achieve CVS NPs. The Fourier transform infrared spectrum confirms the successful modification of DSPE-PEG-TPP on Cu_3_VS_4_ NPs (fig. S2A). The results of thermogravimetric analysis (TGA) suggest that the proportion of DSPE-PEG-TPP in CVS is approximately 31.63% (fig. S2B). CVS NPs display a similar morphology as Cu_3_VS_4_ NPs with good dispersion and no obvious agglomerate (fig. S3A). Furthermore, as confirmed by dynamic light scattering (fig. S3B), the hydrodynamic size of CVS NPs in phosphate-buffered saline (PBS) is approximately 20.31 nm [polydispersity index (PDI) = 0.09 ± 0.06] with a positive charge of ~7.27 mV (fig. S4). In addition, the average size and the PDI of CVS NPs in PBS and RPMI 1640 + 10% fetal bovine serum over 7 days were evaluated (fig. S5). The CVS NPs exhibit a uniform hydrated particle size and demonstrate no obvious PDI variation in various solutions, confirming the excellent stability of CVS NPs in physiological conditions.

### Photothermal performance of CVS NPs

**Fig. 3.**
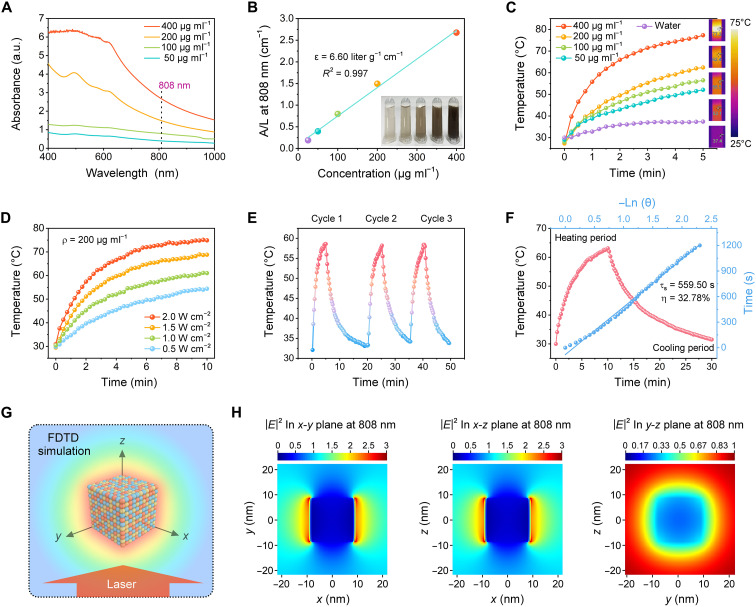
Photothermal evaluation and FDTD simulation of CVS NPs. (**A**) UV-vis-NIR absorbance spectra of the CVS NPs with different concentrations (50, 100, 200, and 400 μg ml^−1^). (**B**) The fitting curve of the mass extinction coefficient of CVS NPs at 808 nm. Inset: Digital photographs of various concentrations of CVS aqueous solution. (**C**) Concentration-dependent photothermal heating curves and corresponding infrared thermal images of CVS aqueous solutions under 808-nm laser irradiation (1.5 W cm^−2^) for 5 min. (**D**) Photothermal heating curves of dispersed CVS NPs (200 μg ml^−1^) under 808-nm laser irradiation at varied power densities. (**E**) Photothermal heating and natural cooling cycles of CVS NPs. (**F**) Photothermal heating curve of CVS NPs (100 μg ml^−1^) under laser irradiation (808 nm, 1.5 W cm^−2^) and cooling curve after turning off the laser (red line). The blue line shows the linear relationship between −lnθ and the time obtained from the cooling. (**G**) Schematic of the 3D FDTD simulation. (**H**) FDTD simulated spatial distribution of the electric field enhancement around the Cu_3_VS_4_ nanostructure illuminated by a 808-nm laser. a.u., absorbance units.

The optical properties of the as-synthesized CVS NPs were evaluated using ultraviolet (UV)–visible (vis)–NIR absorbance spectrum (Fig. 3A), in which the adsorption intensity increased linearly with an elevation of the CVS NPs concentration in the NIR region and the extinction coefficient at 808 nm is determined as 6.60 liter g^−1^ cm^−1^ (Fig. 3B). Subsequently, the photothermal performance of the CVS aqueous solution was assessed excited with 808-nm laser. A notable concentration-dependent photothermal effect is observed, and the infrared thermal images of these solutions verify this phenomenon (Fig. 3C). It is noteworthy that the temperature of the CVS suspension (400 μg ml^−1^) increased considerably by 49.7°C upon 5 min of laser excitation (1.5 W cm^−2^). In contrast, there was only a slight increase in the temperature of pure water, indicating the high efficiency of CVS NPs in transforming NIR laser into thermal energy. Furthermore, CVS NPs have a definite dose-dependent behavior that facilitates the regulation of the PTET temperature gradient by adjusting the injected dose (ID). Moreover, a positive correlation was observed between the temperature of the CVS aqueous solution and the power density of laser, with the former increasing steadily as the latter was elevated (Fig. 3D). The stability and photothermal conversion efficiency are key elements in the photothermal conversion process. To further assess the photothermal stability of CVS NPs, the cycling temperature changes of CVS dispersion were recorded during three laser irradiation/natural cooling cycles. The photothermal performance displays negligible variation after three cycles of heating and natural cooling (Fig. 3E). Besides, no notable changes in optical absorption and particle size are observed for CVS NPs exposed to an 808-nm laser for 30 min, clearly indicating their high photostability (fig. S6). The photothermal conversion efficiency (η) of CVS NPs is calculated to be ~32.78% (Fig. 3F). Given that the PA response is produced by transient laser pulse-induced heating followed by thermoelastic expansion, the in vitro PA imaging capability was measured by embedding different concentrations of CVS NPs solution into agar gel cylinders. CVS NPs exhibit a positive concentration-dependent PA contrast (fig. S7), which can serve as a PA contrast agent.

To further reveal the mechanism of photothermal and PA imaging performance of CVS NPs, the spatial distribution of the electric field intensity was simulated by a FDTD simulation theoretically. A schematic diagram based on the FDTD modeling is shown in Fig. 3G, where an 808-nm laser is used as a NIR source incident along the *z* direction. Considerable electric field enhancement was observed on two sides of the Cu_3_VS_4_ nanostructure (Fig. 3H and movie S1) with intensity value |*E*|^2^ ~ 3 (|*E*| = |*E*_local_/*E*_in_|), where the local and incident electric fields are denoted by *E*_local_ and *E*_in_, respectively. Moreover, the steady-state temperature distribution of the Cu_3_VS_4_ nanostructure in water (refractive index = 1.33) under 808-nm laser irradiation was simulated. The Cu_3_VS_4_ nanostructure exhibits a certain enhanced photothermal effect from the combination of heat generated by the sulfide itself and the interaction with *z*-directional electromagnetic waves, with the temperature difference appearing at two ends of the NPs (fig. S8). It is inferred that the high photothermal conversion efficiency of CVS NPs is due to the narrow bandgap and the intermediate band formed by the introduction of V ions. Owing to the transient optical response leads to the ultrafast nonradiative relaxation of carriers excited from the VB to the IB and the subsequent ultrafast thermalization of the NP lattice, which is consistent with previously reported photothermal models of other IB semiconductors (*46*–*48*).

### Thermoelectric characterization and DFT calculations

In the thermoelectric conversion progress, the Seebeck coefficient (*S*) describes the ability of thermoelectric materials to convert a temperature difference into electrical voltage, defined as *S* = −Δ*V*/Δ*T*. To comprehensively gauge the thermoelectric conversion efficiency of thermoelectric materials, the dimensionless figure of merit (ZT) is defined as

$$\mathrm{ZT}=\frac{S^{2}\sigma T}{\kappa }$$ (1)

where *S* represents the Seebeck coefficient, σ denotes the electrical conductivity, *T* is the absolute temperature, and κ represents the thermal conductivity (*49*). An ideal thermoelectric material is expected to combine the thermal properties of the liquid and the electrical properties of a crystal, which is also known as phonon-liquid electron-crystal thermoelectrics. Specifically, the thermoelectric properties can be improved by two strategies: enhancing their power factor (PF = *S*^2^σ) or reducing thermal conductivity to establish a large temperature difference.

Initially, the thermoelectric performance of Cu_3_VS_4_ NPs after spark plasma sintering (SPS) was measured. The electrical transport properties in the temperature region from room temperature (298 K) to 100°C (373 K) are shown in fig. S9. Cu_3_VS_4_ NPs exhibit excellent conductivity with the resistivity ρ as low as 1.58 milliohm·cm at room temperature. The conductivity σ can be calculated by taking the inverse of the resistivity (σ = 1/ρ), resulting in a conductivity of 63.4 × 10^3^ S m^−1^ for Cu_3_VS_4_. It is obvious that the ρ increases smoothly from 300 to 358 K, after which it begins to increase rapidly, with ρ reaching 1.73 milliohm·cm, and σ is calculated to be 57.7 × 10^3^ S m^−1^ at 373 K. The σ of Cu_3_VS_4_ decreases monotonically with increasing temperature, exhibiting a highly degenerate semiconducting transport behavior. The positive value of the *S* indicates the p-type semiconducting behavior of Cu_3_VS_4_, and as the temperature increases from 298 to 373 K, the *S* increases from 20.7 to 31.9 μV K^−1^ (fig. S9C). On the basis of the measured σ and *S*, the calculated PF at room temperature and 373 K are 27.1 and 58.5 μW m^−1^ K^−1^, respectively (fig. S9D). In addition, the thermal transport properties of Cu_3_VS_4_ are presented in fig. S10 (A to C), including specific heat capacity (*C*_P_), thermal diffusivity (*D*), and thermal conductivity (κ). The κ was examined at 298, 313, 335, 357, and 373 K, and the data are shown in fig. S10C. It can be observed that the κ value increases as the temperature rises, all of which are below 0.4 W m^−1^ K^−1^. The κ of Cu_3_VS_4_ is as low as 0.3 W m^−1^ K^−1^ at room temperature, which is extremely low for thermoelectric materials in the relatively low temperature range (fig. S11). The final calculated ZT values are presented in fig. S10D. Because of the moderate *S* and ultralow κ, the ZT value at room temperature is calculated to be 0.027, reaching 0.060 at 373 K.

**Fig. 4.**
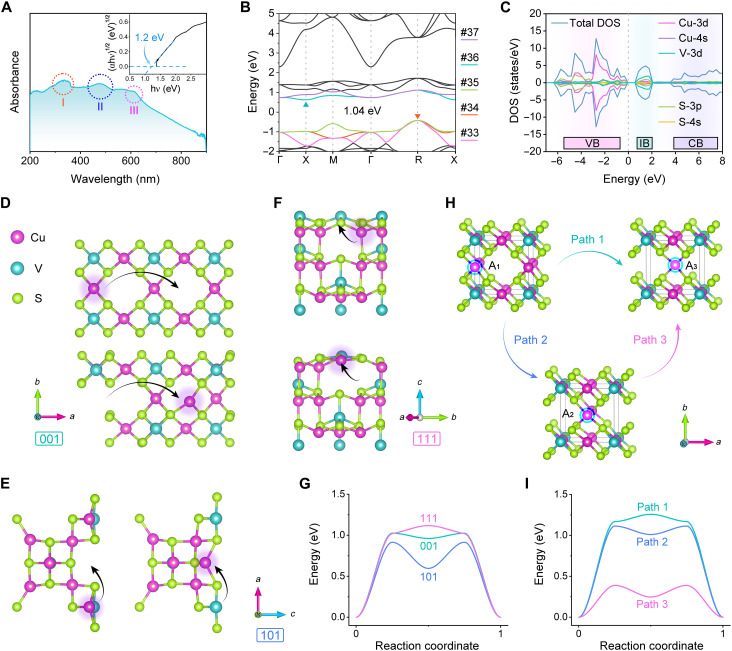
DFT calculations. (**A**) UV-vis diffuse reflectance spectrum of Cu_3_VS_4_. The bandgap estimated by the Tauc plot method is plotted in the inset. (**B**) DFT-calculated band structures of Cu_3_VS_4_ (nonmagnetic system). (**C**) Calculated total and atomic-projected density of states (DOS). The possible diffusion paths of copper ions within (**D**) (001), (**E**) (101), and (**F**) (111) crystal planes are indicated by the arrows. The purple, lake blue, and green balls represent copper atoms, vanadium atoms, and sulfur atoms, respectively. (**G**) Diffusion barriers within different crystal planes. (**H**) Possible paths for the diffusion of copper ions in Cu_3_VS_4_. (**I**) Diffusion barriers in different paths in Cu_3_VS_4_.

To understand the origin of the thermoelectric properties of Cu_3_VS_4_, we further explore its intrinsic structure. It is well known that Cu_3_VS_4_ is an IB semiconductor, whose optical properties are dominated by the collective optical transitions between energy bands. The UV-vis diffuse reflectance spectrum was used to determine the optical properties of the synthesized CVS NPs. There is a broad continuous extinction starting from the NIR region, which extends throughout the visible range (390 to 780 nm; Fig. 4A). Three peaks can be identified and assigned to the electronic transition between states in the VB and the IB (fig. S12). Because of the complexity of the electronic structure of the IB semiconductor, DFT was carried out to calculate the electronic structure of Cu_3_VS_4_ to better understand the electrical transport properties. As can be seen from the energy band structure in Fig. 4B, the top of the VB and the bottom of the IB are situated at the R point and X point in the Brillouin zone, separately, and the calculated indirect bandgap (X → R) is 1.04 eV, which is smaller than the optical measurement result of 1.20 eV (Fig. 4A). One problem with the standard DFT is that it underestimates the electronic bandgap (*50*, *51*). The narrower bandgap of Cu_3_VS_4_ is more favorable for electron transition, leading to higher electrical conductivity for thermoelectric properties. The twofold degeneracy of the IBs (labeled as #36 and #37) and threefold degeneracy of the VBs (#33, #34, and #35) can be observed at the Γ point. Another threefold degeneracy happens at the top of the VB (R point), where they overlap near the Γ and R points because of the inclusion of both heavy bands and light bands. This band overlap, particularly at the bottom of the conduction band (CB)/IB and the top of the VB, is the main physical source for excellent thermoelectric performance (*52*–*54*). Next, the total density of states and the partial density of states of the corresponding band structure of Cu_3_VS_4_ were calculated (Fig. 4C). The overall band structure can be modeled in terms of the VB, IB, and CB, similar to previous theoretical works (*43*, *55*). Obviously, the top of the VB is composed of mainly Cu-3d and S-3p orbitals, while the IB primarily consisted of V-3d states, and the bottom of the CB is an admixture of Cu-4s and S-4s orbitals.

Furthermore, to reveal the diffusion dynamic of Cu ions within the lattice, the NEB method was introduced to estimate the energy barrier. First, the diffusion paths of Cu ions within the (001), (101), and (111) crystal planes were simulated and shown in Fig. 4 (D to F, respectively). The diffusion barriers are calculated to be 1.12, 1.02, and 0.92 eV when the Cu ions diffuse within the (111), (001), and (101) crystal plane, respectively (Fig. 4G). It can be inferred that the migration of Cu ions within (101) is the fastest. Subsequently, we considered three possible pathways when Cu ions migrate within the bulk phase. Because Cu_3_VS_4_ has the cubic phase at room temperature, its crystal lattice symmetry is high, and there are abundant structural equivalent sites for Cu^+^. We hypothesize that Cu^+^ may occupy the midpoint of the unit cell edge (labeled as A_1_), face center (labeled as A_2_), and body center (labeled as A_3_) sites. Path 1 represents the Cu ions at A_1_ sites that migrate directly to A_3_ sites; path 2 represents the transition path from A_1_ to the A_2_ sites, and path 3 refers to the movement of Cu ions from A_2_ to A_3_ sites (Fig. 4H). The diffusion barriers are shown in Fig. 4I; when Cu ions migrate along path 1, a barrier of 1.26 eV can be observed, and the energy potential barrier along path 2 is calculated to be 1.11 eV. It is worth noting that the migration of Cu ions along path 3 is quite fast because the corresponding barrier is only 0.39 eV, much smaller than the values of path 1 and path 2. Our theoretical results fit well with those of Arribart *et al*. These indicated that the compound Cu_3_VS_4_ with cubic phase structure has 3D channels, in which interstitial Cu^+^ ions exhibit a high mobility of 10^−4^ cm^2^ V^−1^ s^−1^ at 300 K (*56*, *57*). Cu ions are able to shift easily from site to site, showing a kinetically disordered state (liquid-like state). The disordered and mobile Cu ions in Cu_3_VS_4_ could not only participate in the conductivity and thus enhance σ but also strongly scatter phonons to decrease the phonon mean free path, resulting in an ultralow thermal conductivity.

### PTE and chemodynamic effects of CVS NPs

**Fig. 5.**
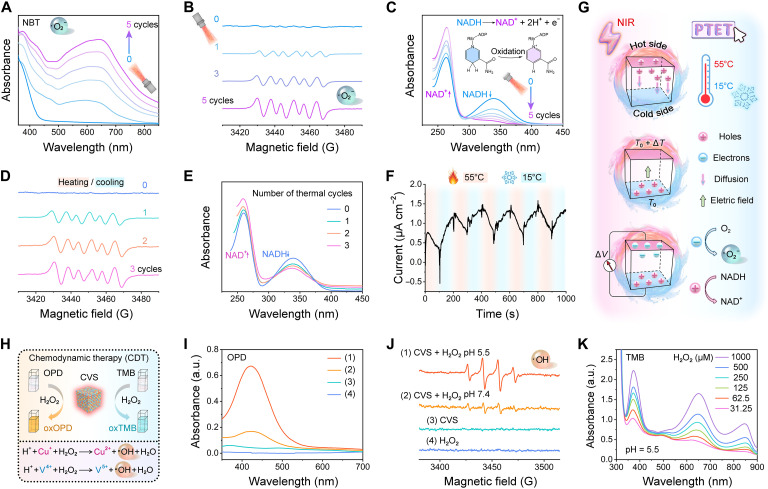
PTE and chemodynamic effects. (**A**) UV-vis absorption spectra of NBT and (**B**) ESR signals for DMPO/**·**O_2_^−^ adducts for five cycles of laser irradiation (1.0 Wcm^−2^). (**C**) PTE oxidation of NADH by CVS NPs during five cycles of laser irradiation. (**D**) ESR spectra detection of DMPO-captured **·**O_2_^−^ during three heating/cooling thermal cycles. (**E**) NADH oxidation during thermal cycles. (**F**) Thermoelectric current of CVS NPs under temperature variations. (**G**) Schematic illustration of the PTET process. (**H**) Schematic illustration of CDT of CVS NPs. (**I**) UV-vis absorption spectra of the catalyzed oxidation of *o*-phenylenediamine (OPD). Group (1) CVS + H_2_O_2_, pH 5.5; (2) CVS + H_2_O_2_, pH 7.4; (3) CVS; and (4) H_2_O_2_. (**J**) ESR spectra of **·**OH trapped by DMPO under different conditions. (**K**) H_2_O_2_ concentration-dependent absorption of 3,3′,5,5′-tetramethylbenzidine (TMB) due to **·**OH generation by CVS NPs.

We next irradiated the CVS NPs with an 808-nm laser for 2 min (laser on), followed by natural cooling to room temperature (laser off). Five laser on/off cycles were executed to research the mechanism of PTET. The detection of **·**O_2_^−^ generated by PTET was performed by nitro blue tetrazolium (NBT) assay, as NBT molecules could capture **·**O_2_^−^ and generate blue-violet NBT-formazan with a characteristic peak at ~560 nm (fig. S13). The characteristic absorption peak is gradually enhanced as the number of cycles increases (Fig. 5A). The electron spin resonance (ESR) spectra confirmed the characteristic sextuplet peaks matching the **·**O_2_^−^ species by 5,5-dimethyl-1-pyrroline *N*-oxide (DMPO) trapping in methanol (Fig. 5B), further indicating that CVS NPs could produce more **·**O_2_^−^ after laser irradiation through the PTE effect. Moreover, the band edge position of Cu_3_VS_4_ was determined using high-resolution VB XPS spectrum. The VB maxima position is located at 0.72 eV (fig. S14A), and the CB minima position is calculated on the basis of the equation *E*_CB_ = *E*_VB_ − *E*_g_, where *E*_g_, *E*_CB_, and *E*_VB_ reveal the bandgap energy, CB potential, and VB potential, respectively. Considering the bandgap of Cu_3_VS_4_ as 1.20 eV, the CB potential is calculated to be −0.48 eV. As shown in fig. S14B, the obtained CB potential is more negative than the redox potential of O_2_/**·**O_2_^−^ [−0.33 eV versus normal hydrogen electrode (NHE)]. The more negative CB potential suggests that the formation of **·**O_2_^−^ radical is possible.

NADH and NAD^+^, as vital cofactors of intracellular redox metabolism, participate in the OXPHOS pathway. The PTE oxidation of NADH was monitored via the UV-vis absorption spectrum. The characteristic absorption peak of NADH (~340 nm) decreases obviously with the increase of the NAD^+^ absorption peak (~260 nm; Fig. 5C), which was attributed to the conversion of NADH to NAD^+^, indicating that CVS NPs could induce the oxidation of NADH under laser on/off cycles. For thermoelectric materials with low thermal conductivity, rapid temperature change (d*T*/d*t*) could lead to a remarkable temperature gradient, which may induce a thermoelectric signal. Therefore, to maximize the thermoelectric effect, in addition to the superior photothermal conversion capability of the material, an additional cooling source is required for a sharp temperature drop to ensure a high d*T*/d*t* rate (*58*). On the basis of this, we performed heating/cooling thermal cycles, in which the temperature variation was alternately manipulated by a hot-water bath (55°C) and a cold-water bath (15°C) apparatus. The CVS solution was wrapped in tin paper to avoid interference from external light. The characteristic sextuplet signal of the DMPO/**·**O_2_^−^ adducts (Fig. 5D) and the oxidation of NADH into NAD^+^ were observed during three thermal cycles (Fig. 5E), demonstrating that temperature changes play an important role in **·**O_2_^−^ generation and NADH oxidation progress. To investigate the generation of thermoelectric charges, we measured the current response of CVS NPs under temperature fluctuation. The current under heating/cooling thermal cycles varies periodically in a temperature-dependent manner (Fig. 5F), indicating the thermoelectric effect of CVS NPs.

As all the above results, CVS NPs could generate **·**O_2_^−^ and induce the oxidation of NADH through the PTE effect under laser irradiation, and its probable mechanism is shown in Fig. 5 (G and H). After the absorption of NIR photons on one side of the CVS NPs, a temperature difference (Δ*T*) is built up through the photothermal conversion progress. The holes (h^+^) at the hot side of the p-type conductor migrate to the cold side because of their increased kinetic energy, and when enough holes are gathered at the cold side, a built-in electric field internal is formed in CVS. With the accumulation of holes, the built-in electric field is gradually enhanced and further prevents the diffusion of holes from the hot side to the cold side. When the carriers inside the NPs reach equilibrium, an electric potential difference (Δ*V*) is formed between the hot and cold sides of the CVS NPs. The excited electron-hole pairs at the temperature gradient could be effectively separated and reached the surface of CVS NPs to further participate in redox reactions, where electrons can be transferred to molecular oxygen generating **·**O_2_^−^, and NADH is oxidized to NAD^+^ by holes.

Because of the variable-valence V and Cu ions, we wondered whether CVS NPs would have a Fenton-like activity for CDT. The generation of **·**OH from H_2_O_2_ decomposition was first examined using the *o*-phenylenediamine (OPD) assay, according to the fact that OPD can be oxidized by the highly reactive **·**OH to give an orange color with the characteristic peak of oxidized-OPD (oxOPD) at ∼420 nm. Neither H_2_O_2_ nor CVS alone had a detectable effect on the absorbance increase of OPD (Fig. 5I). Because the TME is known to be acidic, CVS NPs were expected to function as an intelligent **·**OH generator. Under mildly acidic condition (pH = 5.5), CVS NPs induce a remarkable enhancement in oxOPD characteristic absorption, whereas only a low absorption peak is displayed under neutral conditions (pH = 7.4). Furthermore, ESR spectroscopy experiments affirm the higher production of **·**OH under mildly acidic conditions, as evidenced by the distinct quadruple resonance peaks with a signal intensity ratio of 1:2:2:1 observed in the group (1) using DMPO as the spin trap (Fig. 5J), a characteristic DMPO/**·**OH adduct. Meanwhile, under the weakly acidic TME (pH 5.5), CVS NPs could catalyze the oxidation of 3,3′,5,5′-tetramethylbenzidine (TMB) with the existence of H_2_O_2_ to generate blue oxidized TMB (oxTMB). The absorbance value of oxTMB at ~652 nm increased observably with the increasing H_2_O_2_ concentration (Fig. 5K), thus validating the H_2_O_2_ concentration-dependent chemodynamic performance of CVS NPs. To further clarify the underlying mechanism, XPS analysis was performed to determine the chemical states of V and Cu ions in the CVS NPs after incubation in an acidic H_2_O_2_ environment, and the contents of V^5+^ and Cu^2+^ ions increased compared with that before incubation (fig. S15). The acid-mediated generation of **·**OH by CVS NPs could be ascribed to the oxidation of the lower oxidation state V^4+^/Cu^+^ to V^5+^/Cu^2+^ by H_2_O_2_ in the presence of H^+^. These results indicate that CVS NPs could act as a fascinating Fenton-like agent to produce **·**OH for efficient CDT in an acidic environment. Together with the above excellent chemodynamic performance, the CVS NPs are expected to be used for ROS-based synergistic PTE/CDT.

### Subcellular localization

**Fig. 6.**
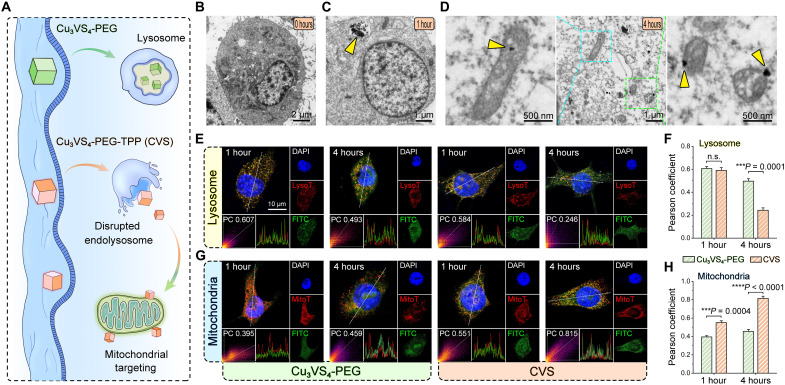
Subcellular localization. (**A**) Schematic illustration of intracellular distribution of CVS NPs after endocytosis. (**B** to **D**) Bio-TEM images of 4T1 cells incubated with CVS NPs for (B) 0 hours, (C) 1 hour, and (D) 4 hours. Yellow arrows suggest the CVS NPs in mitochondria. (**E**) CLSM images of the colocalization of FITC-labeled Cu_3_VS_4_-PEG NPs and FITC-labeled CVS NPs with lysosome and (**F**) corresponding quantification analysis. Statistical analysis was performed via unpaired two-tailed Student’s *t* test. ***P < 0.001; n.s., no significance. Data are presented as means ± SD (*n* = 3). (**G**) Typical CLSM images of the colocalization of FITC-labeled Cu_3_VS_4_-PEG NPs and FITC-labeled CVS NPs with mitochondria and (**H**) corresponding quantification analysis. Statistical analysis was performed via unpaired two-tailed Student’s *t* test. ***P < 0.001 and ****P < 0.0001. Data are presented as means ± SD (*n* = 3). DAPI, 4′,6-diamidino-2-phenylindole.

Previous studies have shown that NPs enter the cells via the endocytic pathway. Afterward, the internalized NPs are usually transported to endosomes/lysosomes, after which they escape from endolysosomes for subsequent intracellular targeting. Thus, lysosomes are the decisive barrier for effective mitochondrial targeting within the intracellular space. Previous studies have shown that NPs enter the cells via the endocytic pathway. Afterward, the internalized NPs are usually transported to endosomes/lysosomes, after which they escape from endolysosomes for subsequent intracellular targeting (*59*, *60*). Thus, lysosomes are the decisive barrier for effective mitochondrial targeting within the intracellular space. Because of the negative charge of the cells and mitochondrial membranes, triphenylphosphine (TPP), an off-domain lipophilic cation, is commonly used to mediate tumor drugs to overcome the barrier hindrance of the cellular and mitochondrial membranes and ultimately target mitochondria (*61*). DSPE-PEG and DSPE-PEG-TPP molecules were coupled to the surface of Cu_3_VS_4_ NPs to form Cu_3_VS_4_-PEG NPs and CVS NPs, respectively, in an attempt to explore whether TPP modification could enhance lysosomal escape and induce mitochondrial accumulation (Fig. 6A). Bio-TEM analysis of 4T1 cells incubated with CVS NPs (Fig. 6, B to D) revealed that CVS NPs were located in endosomes/lysosomes after 1 hour of internalization and targeted accumulation in mitochondria after 4 hours. Furthermore, confocal laser scanning microscopy (CLSM) was used to probe NP intracellular transport. Lysosomes were stained with LysoTracker to assess the endosomal escape ability of NPs. Results show that within the first hour after internalization, both fluorescein isothiocyanate (FITC)–labeled Cu_3_VS_4_-PEG NPs and CVS NPs were colocalized with lysosomes, with Pearson’s correlation coefficients of 0.607 and 0.584 (Fig. 6, E and F). After 4 hours of incubation, CVS NPs were gradually separated from the signal of LysoTracker (Pearson’s correlation of 0.246), indicating their ability to escape from endolysosomes through the proton sponge effect. We investigated the ability of CVS NPs to actively accumulate into mitochondria after the lysosomal escape. Costaining of mitochondria using Mito-Tracker Red FM revealed that the green fluorescence of FITC-labeled Cu_3_VS_4_-PEG NPs barely overlapped with the red fluorescence of mitochondria. In contrast, FITC-labeled CVS NPs clearly overlapped with mitochondria and merged into yellow fluorescence, demonstrating preferential mitochondrial accumulation (Fig. 6G). Quantitative analysis shows that the Pearson coefficient of mitochondria with CVS NPs after 4 hours of incubation is 0.815, much higher than that of Cu_3_VS_4_-PEG NPs at 0.459 (Fig. 6H). These results demonstrate the ability of CVS NPs to overcome cell membrane and solute barriers to specifically target mitochondria, highlighting their potential for PTET.

### In vitro anticancer performance of CVS NPs

**Fig. 7.**
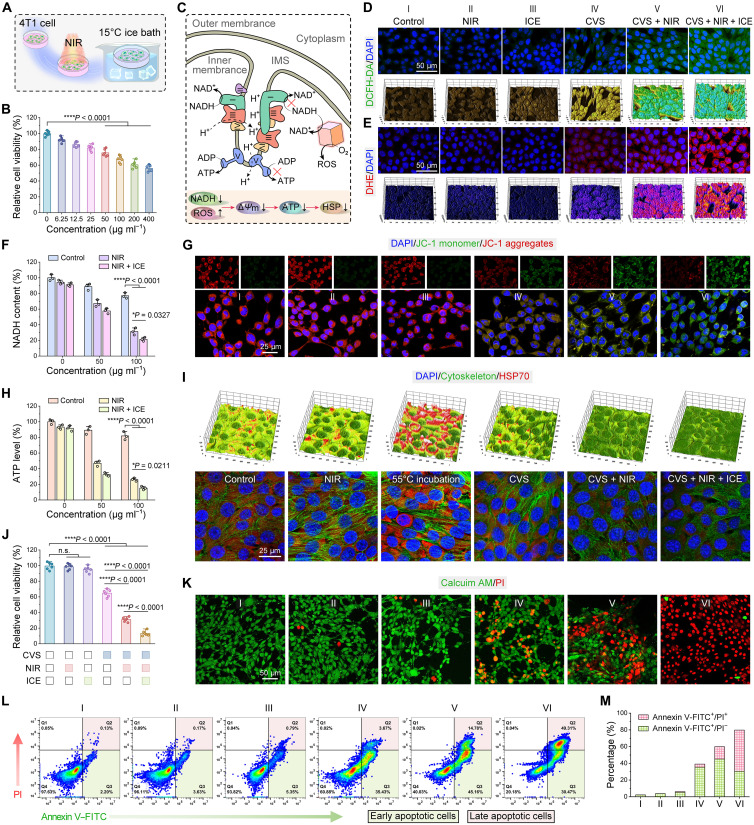
In vitro anticancer performance of CVS NPs. (**A**) Schematic illustration of the heating/cooling process through successively irradiated by an 808-nm laser and cooled by an ice bath. (**B**) Cytotoxicity assays of 4T1 cells with various concentrations of CVS NPs. Data are indicated as means ± SD (*n* = 6). Statistical analysis was executed via one-way analysis of variance (ANOVA) with Tukey’s multiple comparisons post hoc test. ****P < 0.0001. (**C**) Illustration of the inhibitory pathway of HSPs. CLSM images and corresponding 3D surface plot images of intracellular (**D**) ROS and (**E**) **·**O_2_^−^ level with different treatments. (**F**) Quantitative analysis of intracellular NADH content with different treatments. Statistical analysis was performed via one-way ANOVA with Tukey’s multiple comparisons post hoc test. *P < 0.05 and ****P < 0.0001; Data were presented as means ± SD (*n* = 3). (**G**) JC-1 staining of 4T1 cells receiving various treatments. (**H**) Cellular ATP level in 4T1 cells with different treatments. (**I**) Immunofluorescence images of 4T1 cells stained with CoraLite488-conjugated β-actin monoclonal antibody (green) and anti-HSP70 antibody (red) after different treatments. (**J**) Cytotoxicity assays of 4T1 cells with various treatments. (**K**) Calcein AM/PI double staining of 4T1 cells in different groups. (**L**) Flow cytometry analysis of the apoptosis of 4T1 cells treated with various formulations and (**M**) corresponding quantitative analysis of cell apoptosis percentages.

Encouraged by the prominent PTE effect and robust Fenton-like catalytic performance, we further investigated whether it has a potential therapeutic effect on cancer cells. The excellent photothermal effect of CVS NPs at cellular level was confirmed under 808-nm laser irradiation (fig. S16). In addition, an ice pack was used as the cold source to control the temperature to quickly drop to 15°C, thus inducing a sharp temperature drop to ensure a high d*T*/d*t* rate for realizing a higher thermoelectric effect (Fig. 7A). After confirming the negligible cytotoxicity of CVS NPs against RAW 264.7 cells using the methyl thiazolyl tetrazolium (MTT) assay (fig. S17), the cytotoxicity of CVS NPs with different concentrations was further estimated on 4T1 cells. The cell viabilities markedly decline in a dose-dependent manner, and the inhibition rate is 45.83% at 400 μg ml^−1^ (Fig. 7B), probably attributing to the CDT treatment effect. In mitochondria, electrons are transferred to transmembrane protein complexes in the inner mitochondrial membrane, referred to as the electron transport chain (ETC), to produce ATP. A proton gradient across the mitochondrial membrane is created as electrons move through ETC complexes I through IV. Protons then flow back to the mitochondrial matrix from inside the intermembrane space to drive ATP synthesis (Fig. 7C). To investigate the intracellular ROS-generation potential of CVS NPs, we used 2′,7′-dichlorodihydrofluorescein diacetate (DCFH-DA) to evaluate the production of ROS (Fig. 7D). The CVS + NIR + ice group exhibits stronger green fluorescence, illustrating that the production of toxic ROS is attributed to the PTE effect and Fenton-like effect. We assessed the **·**O_2_^−^ content of 4T1 cells in various treatment groups using dihydroethidium (DHE) as an **·**O_2_^−^ probe. Notably, the CVS + NIR + ice group displays the highest red fluorescence intensity compared to other groups, confirming the efficient **·**O_2_^−^ production (Fig. 7E). We wondered whether the CVS NPs could catalyze the oxidization of NADH into NAD^+^, resulting in reduced ATP synthesis and down-regulation of the HSP70 expression. The intracellular NADH content shows concentration-dependent consumption tendencies after heating/cooling treatments (Fig. 7F), indicating that CVS NPs severely consume NADH through the PTE effect. Because of the critical role of mitochondria in the regulation of cancer cell apoptosis, we detected mitochondrial membrane potential (ΔΨm) using JC-1, which is a fluorescent dye sensitive to mitochondrial membrane potential. Compared with the control and NIR groups, 4T1 cells treated with CVS + NIR + ice present evident green fluorescence (Fig. 7G), suggesting that the mitochondrial membrane potential is largely depolarized. Moreover, after being incubated with CVS NPs and then subjected to a heating/cooling process, the cellular ATP secretion is severely suppressed, with a decline of approximately 84.3% with the concentration of 100 μg ml^−1^ (Fig. 7H). The results obtained suggest that CVS NPs with PTE effect could effectively consume the substrate of complex I in the ETC process, thus greatly reducing the production of ATP. Inspired by the satisfactory inhibition performance of ATP generation, we further examined the levels of HSP70 in 4T1 cells treated with various conditions including (i) control, (ii) NIR, (iii) 55°C incubation, (iv) CVS, (v) CVS + NIR, and (vi) CVS + NIR + ice via immunofluorescence assay to evaluate the impact of ATP level on the expression of HSP70 (Fig. 7I). Contrasted with the control and NIR groups, the expression of HSP70 is markedly increased after incubation at a defined heating temperature (~55°C), verifying that when cells are subjected to a heat shock, HSP70 protein is up-regulated. Conversely, the relative HSP70 level of the CVS + NIR + ice–treated group markedly decreased compared with other groups. A similar decreasing tendency of HSP70 was confirmed by Western blotting assay (fig. S18). The cytotoxicity of various formulations was further determined using the MTT assay. As we can see from Fig. 7J, the inhibition rate of the CVS + NIR + ice group is 88.16%, indicating the remarkable synergistic therapeutic efficacy of CDT, HSP inhibition–enhanced PTT, and PTET. The anticancer performance was further tested using the Calcein AM and propidium iodide (PI) double-staining assay (Fig. 7K). An annexin V–FITC and PI double staining assay was carried out to investigate the ability of CVS NPs to induce cell apoptosis. The apoptotic ratio induced by the group of CVS + NIR + ice, 79.78% (sum of Q2 + Q3), which is evidently higher than those of NIR (3.80%), CVS (39.08%), and CVS + NIR (59.94%) under the identical condition (Fig. 7, L and M). The above results provide compelling evidence that CVS NPs can trigger efficient cell apoptosis. To reveal the underlying molecular mechanism of in vitro superior therapeutic effect of CVS NPs, apoptosis-related proteins including Bax, Bcl-2, pro–caspase-3, and cleaved caspase-3 in 4T1 cells were further evaluated by Western blot experiment (fig. S19). Compared with control and NIR-only groups, the expression of proapoptotic Bax protein is increased, and the antiapoptotic Bcl-2 is markedly suppressed, accompanied by cleaved–caspase-3 activation in the CVS + NIR + ice group. These results reflect that the synergistic therapeutic effect of CVS NPs induces apoptosis in 4T1 cells via the intrinsic mitochondria-mediated apoptotic pathway.

### In vivo therapeutic performance of CVS NPs

**Fig. 8.**
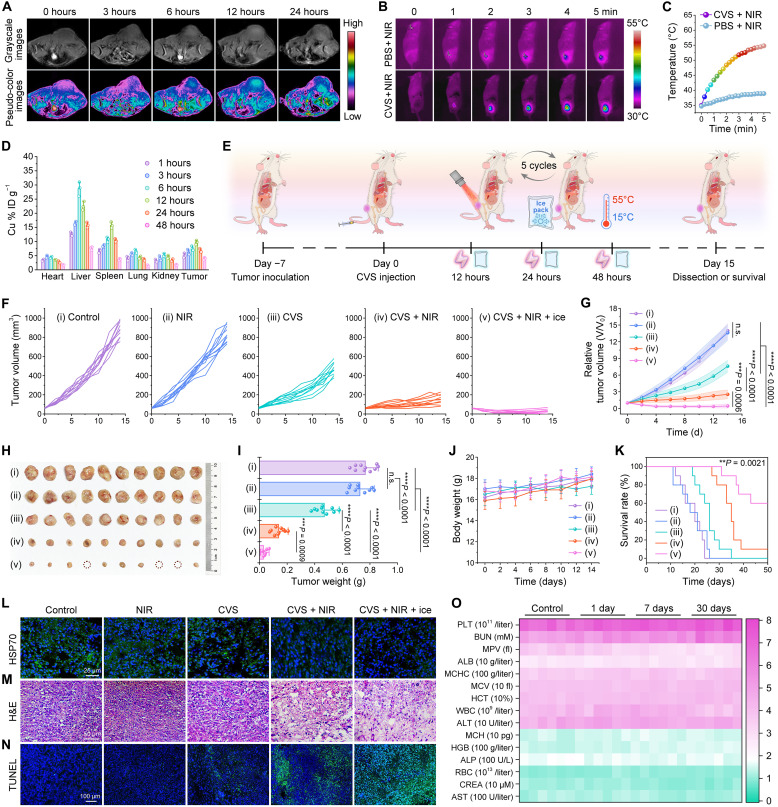
In vivo therapeutic performance of CVS NPs. (**A**) *T*_1_-weighted MR imaging of 4T1 tumor–bearing BALB/c mice intravenously injected with CVS NPs at various time points. (**B**) Infrared thermal images and (**C**) corresponding tumor temperature change of 4T1 tumor–bearing mice after different treatments via the tail vein exposed to an 808-nm laser. (**D**) Biodistribution of Cu (% ID of Cu per gram of tissue) after intravenous injection in main tissues and tumors on different days. Data are indicated as means ± SD (*n* = 3 biologically independent mice per group). (**E**) Schematic illustration of animal therapy by CVS NPs. (**F**) Tumor volume, (**G**) relative tumor volume, (**H**) photographs of excised tumors, and (**I**) average weights of tumors after treatment: (i) control, (ii) NIR, (iii) CVS, (iv) CVS + NIR, and (v) CVS + NIR + ice. Statistical analysis was performed via one-way ANOVA with Tukey’s multiple comparisons post hoc test. ***P < 0.001 and ****P < 0.0001. Data are presented as means ± SD (*n* = 10 biologically independent mice per group). (**J**) Body weight variations and (**K**) Kaplan-Meier survival curves of 4T1 tumor–bearing mice subjected to different treatments (*n* = 10). Asterisks indicate **P = 0.0021 for the CVS + NIR + ice group compared with the control group. (**L**) Expression of HSP70 in tumor regions was evaluated by immunofluorescent staining after different treatments. (**M**) Hematoxylin and eosin (H&E) staining and (**N**) terminal deoxynucleotidyl transferase–mediated deoxyuridine triphosphate nick end labeling (TUNEL) staining of the tumor tissues receiving various treatments. (**O**) Hematological indexes and biochemical data of mice after intravenous injection with PBS and CVS NPs for 1, 7, and 30 days (*n* = 6 mice per group).

Considering that the diamagnetic Cu(I) (3d orbitals: 3d^10^4s^0^) can be oxidized to paramagnetic Cu(II) (3d orbitals: 3d^9^) by H_2_O_2_ on the surface of CVS NPs, there is no doubt that CVS NPs can serve as a suitable agent for TME-responsive *T*_1_-weighted magnetic resonance (MR) imaging. A concentration-dependent signal enhancement effect with the longitudinal relaxation (*r*_1_) value of 4.07 mM^−1^ s^−1^ is observed after incubation with H_2_O_2_, higher than that of CVS NPs without H_2_O_2_ addition (*r*_1_ = 0.41 mM^−1^ s^−1^; fig. S20). Thereafter, time-dependent MR imaging capability was evaluated in 4T1 tumor–bearing mice after intravenous injection of CVS NPs at different time intervals (0, 3, 6, 12, and 48 hours). The *T*_1_-MR signals of the tumor are markedly enhanced with the injection time extension, whose intensity reaches a maximum level after 12 hours of intravenous injection (Fig. 8A), suggesting the efficient tumor-homing and in situ self-enhancement MR imaging capacities. In view of the intense PA effect and considerable photothermal properties of CVS NPs in vitro, we then examined their performance in real-time PA imaging of localized tumors in vivo. After intravenous injection of CVS NPs into the 4T1 tumor–bearing mice, PA images of the tumor region were recorded at 0, 3, 6, 12, 24, and 48 hours after injection, respectively. The PA signals at the tumor site gradually increased (fig. S21A) and reached a maximum after 12 hours of administration, demonstrating a 4.8-fold increase contrasted with preinjection (0 hours; fig. S21B). It is indicated that CVS NPs have excellent PA imaging performance for therapeutical guidance. Furthermore, a thermal imaging camera was used to monitor the real-time temperature of the tumor site at 12 hours after injection of CVS NPs. Under 808-nm laser (1.0 W cm^−2^) irradiation, the temperature of the tumor can be raised to 54.8°C within 5 min (Fig. 8, B and C). As a comparison, the control group only shows a temperature increase of about 4.2°C at the tumor site, suggesting that CVS NPs also have a superior NIR-photothermal effect in vivo. Moreover, the inductively coupled plasma−optical emission spectrometry measurement was used to comprehensively analyze the in vivo behavior of CVS NPs after intravenous injection into 4T1 tumor–bearing mice. As shown in Fig. 8D, high levels of Cu are observed in the liver, spleen, lung (reticuloendothelial system), and kidney. At 12 hours after injection of CVS NPs, Cu ions in the tumor regions reach a maximum concentration of 9.71% ID g^−1^ and maintain a relatively high content of 4.01% ID g^−1^ even after 48 hours, indicating superior tumor-homing efficiency of CVS NPs. As displayed in fig. S22A, the blood circulation half-life time of CVS NPs is calculated to be *t*_1/2_(α) = 0.24 hours and *t*_1/2_(β) = 3.75 hours. The moderate circulation time is beneficial to the accumulation of NPs in the tumor. High levels of Cu could be detected in feces of mice after intravenous injection of CVS NPs (fig. S22B), indicating that CVS NPs are easily excreted through fecal and renal pathways, thus avoiding the long-term toxicity of the NPs in mice.

To further investigate the in vivo antitumor efficacy of CVS NPs, a 4T1 tumor–bearing female BALB/c mice xenograft model was established by subcutaneously injecting 4T1 cells (2 × 10^6^) on the right back of each mouse (Fig. 8E). Twenty-five 4T1 tumor–bearing mice were randomly divided into the following groups (*n* = 10 per group) and treated accordingly: (i) control (PBS), (ii) NIR, (iii) CVS NPs, (iv) CVS NPs + NIR, and (v) CVS NPs + NIR + ice pack (five cycles). For mice in groups (iii) to (v), CVS NPs (100 μl, 15 mg kg^−1^) were firstly injected intravenously, where group (iii) was not further treated, group (iv) was subjected to an 808-nm laser (1.0 W cm^−2^), while group (v) was successively irradiated with a 808-nm laser and cooled by an ice pack for five cycles at 12, 24, and 48 hours after injection. Notably, an ice pack was used as the cold source to control the temperature to quickly drop to 15°C, thus inducing a sharp temperature drop to ensure a high d*T*/d*t* rate. The real-time temperature at the tumor region in the whole heating/cooling process was observed using a thermal imaging camera. Next, the tumor growth profiles were monitored throughout the entire treatment, as illustrated in Fig. 8 (F and G), the CVS + NIR + ice group achieves the most remarkable regression with a suppression rate of 98.28%, while no conspicuous inhibitory effect on tumor growth is observed in control groups, which is further confirmed by measuring the weights of the dissected tumor tissue and the corresponding visualized photographs (Fig. 8, H and I).

These results collectively suggest that the CVS NPs treated with heating/cooling cycles can greatly suppress the growth of tumors mainly attributing to the cooperative effect of CDT, HSP inhibition–enhanced PTT, and PTET as follows: (i) The CVS NPs could convert endogenous H_2_O_2_ into ·OH via a typical Fenton-like reaction. (ii) CVS NPs could catalytically generate **·**O_2_^−^ and induce the oxidation of endogenous NADH through PTE effect, which decrease the secretion of ATP during treatment to further down-regulate the expression of HSP70. Furthermore, the body weights of mice in each group were recorded every 2 days throughout the 14-day assessment period. There was no notable difference in body weights of mice in each group, which displays that the injection of CVS NPs has no apparent systemic toxicity (Fig. 8J). The mice receiving CVS + NIR + ice treatment show the longest lifetime without any tumor recurrence, indicating that the synergistic treatment can obviously improve the survival rate of tumor-bearing mice (Fig. 8K). To further understand the mechanism of synergistic therapy, DCFH-DA staining and DHE staining of tumor slices were performed to assess the production of ROS and **·**O_2_^−^ in the tumor after different treatments (fig. S23). Contrasted with the weak fluorescence in tumor slices from control, NIR, and CVS groups, the CVS + NIR group shows an obvious fluorescence signal, and the strongest fluorescence is observed in the CVS + NIR + ice group, suggesting the efficient production of ROS and **·**O_2_^−^. Subsequently, a commercial NADH/NAD^+^ assay kit was used to evaluate NADH/NAD^+^ redox ratios in tumor regions of mice (fig. S24), and the expression level of HSP70 in different groups was examined using an immunofluorescence assay (Fig. 8L). In the CVS + NIR + ice group, evident decreases in HSP70 levels and NADH/NAD^+^ ratio were observed, confirming that the holes generated in the heating/cooling process can catalyze the oxidization of the NADH to NAD^+^ to inhibit the synthesis of ATP. In addition, the hematoxylin and eosin staining and terminal deoxynucleotidyl transferase–mediated deoxyuridine triphosphate nick end labeling staining were performed on tumor sections from different groups to further acquire insight into the therapeutic effect of the CVS NPs (Fig. 8, M and N). In accordance with the tumor growth inhibition results, the CVS + NIR + ice group presents the most severe tumor impairment and the increased population of apoptotic cells among all groups. To evaluate the biocompatibility of the CVS NPs for potential practical applications, blood routine and blood biochemical analyses were carried out on healthy BALB/c mice after intravenous injected with CVS NPs. No apparent abnormalities of liver and kidney function can be identified before and after the intravenous administration of CVS NPs (Fig. 8O). Throughout the evaluation period, all hematological biomarkers show no noticeable differences contrasted with the control group, indicating that CVS NPs have negligible side effects on the hematological system. In addition, the normal organs of mice in various groups have no obvious inflammation or damage after 15 days of treatment (fig. S25). In general, all these results demonstrate that the obtained CVS-PTE-nanocatalysts have satisfactory biosecurity of CVS NPs for possible in vivo therapeutic applications.

## DISCUSSION

In this proof-of-concept study, we introduced phonon-liquid CVS NPs, which have a prominent PTE effect to overcome the restrictions of conventional thermoelectric therapy, including low thermoelectric catalytic efficiency at human body temperature and inevitable tumor thermotolerance. The thermal and electrical transport properties of Cu_3_VS_4_ nanostructure were calculated by DFT calculations: a threefold degeneracy at the top of the VB and a relatively low diffusion barrier of Cu ions within the lattice, resulting in an ultralow thermal conductivity of 0.3 W m^−1^ K^−1^ at room temperature. The obtained CVS NPs exhibit excellent photothermal conversion efficiency (32.78%) and ideal PA imaging capability exposed to an 808-nm laser. Meanwhile, the electrons and holes excited by the built-in thermoelectric field can effectively engage in redox reactions on the surface of CVS NPs, generating **·**O_2_^−^ and oxidizing NADH into NAD^+^. Notably, the depletion of endogenous NADH through the PTE effect triggers a domino effect: The function of mitochondrial respiratory complex I can be competitively inhibited and substantially leads to impairment of OXPHOS, cutting off the ATP supply and eventually causing the down-regulation of HSP70 levels. In addition, in the specific acidic microenvironment of the tumor, CVS NPs produce highly toxic **·**OH for CDT through a Fenton-like reaction with H_2_O_2_. In vivo experiments guided by the designed treatment protocols show that CVS NPs synergistically induced apoptosis, efficiently suppressing tumor growth with a inhibition rate of 98.28% in the CVS plus NIR and ice pack group. Together, this work not only provides a simple but efficient strategy to conquer the limitations of traditional PTT through ROS-based synergistic PTET/CDT but also validates the great potential of mitochondrial metabolism modulation in the field of cancer therapy.

## MATERIALS AND METHODS

### Synthesis of Cu_3_VS_4_ NPs

The synthesis started with the preparation of sulfur precursor. 1-Dithiothreitol (DDT) (12 ml) and oleylamine (OLAM) (5 ml) were mixed in a 50-ml three-neck flask, then dried under vacuum at 110°C for 30 min and heated to 160°C under nitrogen for 30 min, and then cooled naturally. Meanwhile, metal precursors were prepared. Specifically, CuI (0.190 g, 1 mmol), V(acac)_3_ (0.463 g, 1.33 mmol), trioctylphosphine (TOP) (0.446 ml, 1 mmol), and 1-octadecene (ODE) (7 ml) were introduced into another 50-ml three-neck flask and dried under vacuum at 110°C for 30 min through a Schlenk line. The temperature was increased to 280°C with a heating rate of 10°C min^−1^ under a protective atmosphere of nitrogen. Afterward, the sulfur precursor solution (3.4 ml) was rapidly injected into the metal precursor solution. After injection, the temperature was allowed to recover, and the final solution was maintained at the target temperature for 20 min, after which it was cooled down to room temperature. The resulting NPs were washed three times with a mixture of ethanol and hexane in a volume ratio of 1:3 to remove excess ligands and unreacted precursors. The resulting precipitate was dissolved in hexane and stored in a glove box for further studies.

### Surface modification of the NPs

The Cu_3_VS_4_ NPs in hexane were collected by centrifugation at 13,000 rpm for 10 min. Cu_3_VS_4_ NPs (20 mg) and DSPE-PEG-TPP (20 mg) were mixed in dichloromethane (10 ml) under magnetic stirring for 12 hours; then, the solvent of dichloromethane was removed by a rotary evaporator. Then, the as-prepared Cu_3_VS_4_-PEG-TPP NPs (CVS NPs) were obtained after centrifuging three times and washed with deionized water. For the synthesis of FITC-labeled CVS NPs, Cu_3_VS_4_ (20 mg), DSPE-PEG-TPP (20 mg), and DSPE-PEG-FITC (1 mg) were mixed in dichloromethane (10 ml) and stirred in the dark for 12 hours. For FITC-labeled Cu_3_VS_4_-PEG NPs synthesis, Cu_3_VS_4_ (20 mg), DSPE-PEG (20 mg), and DSPE-PEG-FITC (1 mg) were mixed in dichloromethane (10 ml) and stirred in the dark for 12 hours. Other protocols were the same as those described above. The resulting NPs were redispersed in PBS and kept in the dark before use.

### DFT calculation

DFT calculations were carried out using the VASP package (*62*, *63*). With the application of Perdew-Burke-Ernzerhof functional (*64*) and projector augmented wave (PAW) technique (*65*), the plane wave energy cutoff is set to 500 eV. The integration over the Brillouin zone was treated by the (3 × 3 × 3) and (3 × 3× 1) Monkhorst-Pack grid (*66*) for bulk crystal and the surfaces, respectively, and a vacuum slab with a thickness of 15 Å was used during the calculation. The convergence criteria for energy and forces are 10^−5^ eV and −0.01 eV/Å.

### Electrochemical characterization

The thermoelectric current of CVS NPs under temperature variations was measured by an electrochemical analyzer (CHI660E) in the Na_2_SO_4_ aqueous solution (0.5 M) using a Pt plate as the counter electrode and Ag/AgCl as a reference electrode. The working electrode was fabricated by depositing the suspension (20 μl) prepared from CVS NPs (10 mg) mixed with Nafion (1 mM, 2 ml) in ethanol onto a fluorine-doped tin oxide (FTO) glass.

### Oxidation of NADH

To explore the oxidation of NADH during PTET, we irradiated a mixture of CVS NPs solution (100 μg ml^−1^) and NADH solution (50 μg ml^−1^) with an 808-nm NIR laser (1.0 W cm^−2^) for 2 min (laser on), followed by natural cooling to room temperature (laser off) for a total of five laser on/off cycles. The absorbance changes of NADH at 340 nm and NAD^+^ at 260 nm were recorded using a UV-vis spectrophotometer. Subsequently, heating/cooling thermal cycles were performed for 3 ml of the mixed solution of CVS NPs (100 μg ml^−1^) and NADH (50 μg ml^−1^), in which the temperature variations were manipulated alternately by a hot-water bath (55°C) and a cold-water bath (15°C) apparatus. The CVS solution was wrapped in tin paper to avoid interference from external light.

### Evaluation of ROS generation by PTET/CDT

We verified the generation of **·**O_2_^−^ during PTET by NBT assay. Typically, NBT solution (100 μl, 0.01 mM) in dimethyl sulfoxide was mixed with CVS NPs (1 ml, 20 μg ml^−1^) in PBS. Similar to the above-mentioned procedure, five laser on/off cycles were performed, and the absorption at 680 nm was measured on a UV-vis spectrophotometer.

OPD and TMB were used to detect the production of **·**OH by CVS NPs through CDT. Typically, OPD (1.0 mM) and CVS NPs (20 μg ml^−1^) were incubated in PBS solution containing 0.5 mM H_2_O_2_ with pH set to 5.5 or 7.4. For comparison, OPD mixed with CVS NPs or H_2_O_2_ were also detected. The absorbance changes of OPD at 420 nm were recorded. In addition, CVS NPs (20 μg ml^−1^) were treated with various concentrations of H_2_O_2_ (31.25, 62.5, 125, 250, 500, and 1000 μM) in 2 ml of PBS solution (pH 5.5) containing TMB (0.5 mM). After coincubation for 15 min, the absorbance of TMB at 652 nm was monitored to detect the generation of **·**OH.

### ESR measurements

Quantitative analysis of ROS production was performed on an ESR spectrometer setup with a center field of 3450 G using 1.0-mm quartz tubes. DMPO dispersed in methanol and water were used to detect **·**O_2_^−^ and **·**OH, respectively. For the detection of **·**O_2_^−^, CVS NPs (20 μg) were mixed with DMPO (10 μl, 1.0 M) in methanol (1 ml) for five laser on/off cycles or three heating/cooling thermal cycles, similar to above mentioned procedure. To detect **·**OH, CVS NPs (20 μg) and DMPO (10 μl, 1.0 M) were mixed in PBS (1 ml) with H_2_O_2_ (0.5 mM), pH = 5.5 or 7.4. For comparison, DMPO mixed with CVS NPs or H_2_O_2_ were also detected.

### Subcellular localization

The subcellular localization of the NPs was investigated by confocal microscopy. Briefly, 4T1 cells were seeded in a six-well plate containing 28-mm cover glass at 1 × 10^5^ cells each well and cultured overnight for adherence. Then, cells were incubated with a medium (1 ml) containing FITC-labeled Cu_3_VS_4_-PEG NPs (100 μg ml^−1^) or CVS NPs (100 μg ml^−1^) for 1 hour and 4 hours. Subsequently, the cells were stained with commercial LysoTracker Red DND-99 (100 nM) or Mito-Tracker Red FM (100 nM) according to the manufacturer’s guidelines, and then counterstained with 4′,6-diamidino-2-phenylindole (DAPI) for 15 min. After incubation, the 4T1 cells were rinsed with PBS several times and fixed with glutaraldehyde (2.5%). Last, the fluorescence images were recorded using a CLSM, and the colocalization analysis was conducted using the ImageJ software.

### In vitro cytotoxicity evaluation

Cytotoxicity evaluation of CVS NPs was performed on 4T1 mammary cells via the standard MTT assay. 4T1 cells were treated with different concentrations of CVS NPs (0, 6.5, 12.5, 25, 50, 100, 200, and 400 μg ml^−1^) for 24 hours. MTT assay was used to test the cytotoxicity following a standard protocol. The therapeutic effect of CVS NPs was further studied. Briefly, 4T1 cells were treated with (i) control, (ii) NIR, (iii) ice, (iv) CVS, (v) CVS + NIR, and (vi) CVS + NIR + ice. The content of CVS in groups (iv), (v), and (vi) was 100 μg ml^−1^. Groups (ii) and (v) were irradiated with an 808-nm laser (1.0 W cm^−2^) for 10 min. Groups (iii) and (vi) used an ice pack as a cold source in a water bath to control the temperature to drop to 15°C quickly. While group (iv) was under 808-nm laser (1.0 W cm^−2^) irradiation for 2 min and cooled by an ice bath for five cycles. Subsequent treatments were conducted with the same experimental conditions as mentioned above.

### Cell imaging

4T1 cells were seeded into six-well dishes and cultured overnight. Then, the cells were treated with different formulations: (i) control, (ii) NIR, (iii) ice, (iv) CVS, (v) CVS + NIR, and (vi) CVS + NIR + ice. The content of CVS in groups (iv), (v), and (vi) was equivalent. Groups (iii) and (vi) used ice packs as a cold source in a water bath to control the temperature to quickly drop to 15°C. After 4 hours of incubation, group (v) was irradiated by 808-nm laser irradiation (1.0 W cm^−2^) for 10 min, while group (iv) was under irradiation for 2 min and cooled by an ice bath for five cycles.

For in vitro ROS detection, after accepting the above treatments, 4T1 cells were stained with a fluorescent ROS probe (DCFH-DA: 20 μM, 1 ml) for 20 min. The cell fluorescence images were performed by a CLSM (Leica TCS SP8) with 488-nm laser excitation.

For intracellular **·**O_2_^−^ evaluation, after 4T1 cells accepted the above treatments, the culture medium was replaced with DHE staining solution (30 μM) and cultured for another 40 min, followed by the nucleus being counterstained by DAPI for 15 min. Then, the cells were imaged using CLSM.

For mitochondrial integrity assay, after the treatments above, 4T1 cells were incubated with JC-1 staining solution (Beyotime Institute of Biotechnology, C2005) following the manufacturer’s protocol. The cells were then photographed using fluorescence microscopy in the red channel for J-aggregates and green channel for J-monomer, separately.

For immunofluorescence staining, 4T1 cells were treated with (i) control, (ii) NIR, (iii) 55°C incubation, (iv) CVS, (v) CVS + NIR, and (vi) CVS + NIR + ice, followed by fixation in 4% paraformaldehyde for 15 min, and then permeabilized with 0.3% Triton X-100 for 30 min. Nonspecific binding was blocked using 5% bovine serum albumin. The cells were then incubated with primary antibodies of anti-HSP70 for 2 hours, while the cytoskeleton was labeled using CoraLite488-conjugated β-actin monoclonal antibody. After washing three times with PBS, the corresponding Alexa Fluor 555–conjugated anti-rabbit immunoglobulin G secondary antibody was added and incubated for 1 hour. Last, the cell nucleus was stained with DAPI and examined using CLSM.

For live/dead cell staining, after 4T1 cells accepted the above treatments, the treated cells were stained with Calcein AM (2 μM) and PI (4 μM) for 15 min. After fixing the cells with glutaraldehyde and washing them with PBS, all fluorescence images were acquired via CLSM.

### Detection of cellular NADH content

4T1 cells were seeded in six-well plates and incubated for 24 hours. Then, the cells were further incubated with various concentrations of CVS NPs solution (0, 50, and 100 μg ml^−1^) for another 12 hours, respectively. In particular, an 808-nm laser (1.0 W cm^−2^) was used for the CVS + NIR group for 10 min during the incubation. In CVS + NIR + ice group, ice was used as a cold source in a water bath to control the temperature to drop to 15°C quickly, and cells were irradiated by an 808-nm laser (1.0 W cm^−2^) for 2 min and cooled by an ice bath for five cycles. The NADH content of each group was obtained using the NADH/NAD^+^ Assay Kit (Beyotime Institute of Biotechnology) according to the manufacturer’s instructions.

### Evaluation of intracellular ATP level

4T1 cells were seeded each well on a six-well plate (1 × 10^5^ cells per well) and cultured overnight. Fresh medium (1 ml) containing CVS NPs with various concentrations (0, 50, and 100 μg ml^−1^) was added to each well and incubated for 12 hours. Then, an 808-nm laser (1.0 W cm^−2^) was used for the CVS + NIR group for 10 min during the incubation. In the CVS + NIR + ice group, ice was used as a cold source in a water bath to control the temperature to quickly drop to 15°C, and cells were exposed to an 808-nm laser (1.0 W cm^−2^) for 2 min and cooled by an ice bath for five cycles. Then, the ATP level in each group was assessed using the ATP assay kit. Briefly, the cells were collected and lysed in RIPA buffer and centrifuged at 12,000 rpm for 10 min. Then, a supernatant (20 μl) was mixed with ATP detection working solution (100 μl) in opaque-walled 96-well plates, and luminescence was monitored at 560 nm using a luminometer.

### Apoptosis assay

4T1 cells were seeded in a six-well plate at an initial density of 1 × 10^5^ cells per well. After 24 hours of cell adhesion, the cells were treated with the following: (i) control, (ii) NIR, (iii) ice, (iv) CVS, (v) CVS + NIR, and (vi) CVS + NIR + ice. The content of CVS in groups (iv), (v), and (vi) was 100 μg ml^−1^. Groups (ii) and (v) were irradiated with an 808-nm laser (1.0 W cm^−2^) for 10 min. Groups (iii) and (vi) used ice packs as a cold source in a water bath to control the temperature to drop to 15°C. While group (iv) was irradiated by the 808-nm laser (1.0 W cm^−2^) for 2 min and cooled by an ice bath for five cycles. All treated cells were trypsinized, washed, and quantified by an annexin V–FITC/PI apoptosis detection kit by a flow cytometer (BD Accuri C6).

### Western blot assays

4T1 cells were preseeded in a six-well culture plate (1 × 10^5^ cells per well). For HSP assays, the experiment was divided into six groups: (i) control, (ii) NIR, (iii) 55°C incubation, (iv) CVS, (v) CVS + NIR, and (vi) CVS + NIR + ice. For Bax, Bcl-2, pro–caspase-3, and cleaved–caspase-3 assays, the experiment was divided into five groups: (i) Control, (ii) NIR, (iii) CVS, (iv) CVS + NIR, and (v) CVS + NIR + ice. The 4T1 cells were collected using trypsin and lysed in a lysis buffer. Then, the proteins were separated by SDS–polyacrylamide gel electrophoresis gel electrophoresis and then transferred into a polyvinylidene fluoride membrane. After blocking with 5% dried skimmed milk for 1 hour, the samples were stained with the corresponding primary antibody (anti-HSP70 antibody, anti-Bax antibody, anti–Bcl-2 antibody, anti–pro–caspase-3 antibody, and anti–cleaved–caspase-3 antibody) overnight and anti–β-actin antibody as the loading control, followed by cultivation with the goat anti-rabbit Dylight-800 antibody for 1 hour. Afterward, the membrane was visualized by an Odyssey CLx Image Studio.

### Animal experiments

Female BALB/c mice (4 weeks old) were obtained from Beijing Vital River Laboratory Animal Technology Co. Ltd. (Beijing, China). All animal experiments were approved by the Drug Safety Evaluation Center of Harbin Medical University, China (1100111084356). The animals were maintained in a specific pathogen–free condition at 26 ± 1°C and 50 ± 5% humidity with a 12-hour light/12-hour dark cycle, and the procedure of animal experiments was performed under the Guidelines for Care and Use of Laboratory Animals of the Drug Safety Evaluation Center of Harbin Medical University (no. SYDW 2020-051). 4T1 tumor models were established by subcutaneously injecting 4T1 cells (2 × 10^6^) dispersed in PBS (100 μl) on the right back of each BALB/c mouse and used for the following experiments when the tumor volume approached 60 mm^3^.

### In vitro and in vivo MR imaging performance

The in vitro and in vivo MR imaging was measured by an animal MR imaging scanner (BioSpec 94/20USR 9.4 T). CVS NPs were dissolved in PBS solution with different Cu concentrations (0, 0.075, 0.15, 0.3, 0.2, 0.6, and 1.2 mM) with or without H_2_O_2_ in several 1.5-ml centrifuge tubes. The *T*_1_ MR image signal intensity of CVS NPs at various sample concentrations was measured. In vivo *T*_1_-weighted MR imaging experiments were done by intravenously injecting the CVS NP solution (100 μl) with a concentration of 15 mg kg^−1^. Then, the *T*_1_-weighted MR images were obtained before and after 3, 6, 12, and 24 hours of injection.

### In vivo PA imaging performance

Female BALB/c mice bearing 4T1 tumors were injected with CVS NPs (100 μl, 15 mg kg^−1^) through the tail vein. At 0, 3, 6, 12, and 24 hours after injection, the tumor region was imaged using the PA computed tomography system.

### In vivo therapeutic evaluation of CVS NPs

Twenty-five 4T1 tumor–bearing mice were randomly divided into five groups (*n* = 10 per group) as follows: (i) control (PBS), (ii) NIR, (iii) CVS, (iv) CVS + NIR, and (v) CVS + NIR + ice pack (five cycles). Afterward, the body weight and tumor volume were recorded every 2 days, and the tumor volumes (in mm^3^) were calculated according to *V* = *lw*^2^/2, in which *w* and *l* are the width and length of the tumor, respectively. For mice in groups (iii) to (v), CVS NPs (100 μl, 15 mg kg^−1^) were first injected intravenously, where group (iii) was not further treated and group (iv) was subjected to 808-nm laser (1.0 W cm^−2^) irradiation at 12, 24, and 48 hours after injection. Group (v) was successively irradiated by an 808-nm laser for 5 min and cooled by an ice pack for five cycles at 12, 24, and 48 hours after injection. Notably, an ice pack was used as the cold source to control the temperature to drop to 15°C.

### Biochemical analysis of blood

Female BALB/c mice without tumors (*n* = 6) were treated with PBS (as control, 100 μl) or CVS NPs (100 μl, 15 mg kg^−1^) through intravenous injection. After 1, 7, and 30 days of injection, blood samples were collected. For routine blood analysis and blood biochemistry tests, parameters such as platelets, blood urea nitrogen, mean platelet volume, albumin, hematocrit, mean corpuscular volume, mean corpuscular hemoglobin concentration, white blood cell, hemoglobin, alanine transaminase, mean corpuscular hemoglobin, alkaline phosphatase, red blood cells, creatinine, and aspartate aminotransferase were measured.

### Statistical analysis

Unpaired two-tailed Student’s *t* test was used to compare the statistical significance between two data groups. One-way analysis of variance (ANOVA) with Tukey’s multiple comparisons post hoc test was used to compare three or more groups. Quantitative data were indicated as means ± SD. Asterisks were used to represent significant differences (n.s., no significance; *P < 0.05, **P < 0.01, ***P < 0.001, and ****P < 0.0001). The statistical analysis was performed by using GraphPad Prism 9.0 software.

## Supplementary Materials

### This PDF file includes:

Supplementary Text Figs. S1 to S25 Legend for movie S1 References
