# Supplementary material for: Mitochondria-targeting Cu3VS4 nanostructure with high copper ionic mobility for photothermoelectric therapy
Source: Sci Adv. 2023 Nov 1;9(44):eadi9980. doi: 10.1126/sciadv.adi9980 (PMC10619935; doi:10.1126/sciadv.adi9980)
Supplement: Supplementary file 1 — Supplementary Text Figs. S1 to S25 Legend for movie S1 References [file sciadv.adi9980_sm.pdf]

Science Advances
AAAS

Supplementary Materials for

Mitochondria-targeting Cu_3VS_4 nanostructure with high copper ionic mobility for photothermoelectric therapy

Yushan Dong *et al.*

Corresponding author: Lili Feng, fenglili@hrbeu.edu.cn; Piaoping Yang, yangpiaoping@hrbeu.edu.cn; Yanli Zhao, zhaoyanli@ntu.edu.sg

Sci. Adv. **9**, eadi9980 (2023)
DOI: 10.1126/sciadv.adi9980

The PDF file includes:

Supplementary Text
Figs. S1 to S25
Legend for movie S1
References

Other Supplementary Material for this manuscript includes the following:

Movie S1

Supplementary Text

Materials

Copper(I) iodide (CuI, 98%), 5,5-dimethyl-1-pyrroline-N-oxide (DMPO), 1-dodecanethiol (DDT, 98%), elemental sulfur (S, 99.99%), hexane (anhydrous, 95%), nicotinamide adenine dinucleotide (NADH), 1-octadecene (ODE, 90%), oleic acid (OA, 90%), oleylamine (OLAM, 70%), *o*-phenylenediamine (OPD), 3,3',5,5'-tetramethyl-benzidine (TMB), trioctylphosphine (TOP, 90%), and vanadium acetylacetonate ($V(acac)_3$, 97%) were purchased from Aladdin (Shanghai, China). 1,2-Distearoyl-sn-glycero-3-phosphoethanolamine-N-[methoxy(polyethylene glycol)2000]-triphenylphosphonium bromide (DSPE-PEG_{2000}-TPP), DSPE-PEG_{2000}, and DSPE-PEG_{2000}-FITC were obtained from Ruixi Biological Technology Co., Ltd. (Xi'an, China). Adenosine triphosphate (ATP) assay kit, annexin V-FITC/PI apoptosis assay kit, calcein-AM, 2',7'-dichlorofluorescein diacetate (DCFH-DA), dihydroethidium (DHE), H&E, JC-1 staining kit, 4',6-diamidino-2-phenylindole (DAPI), methylthiazolyldiphenyl-tetrazolium bromide (MTT), NAD^+/NADH assay kit, propidium iodide (PI), and TUNEL apoptosis assay kit were acquired from Beyotime Institute of Biotechnology (Haimen, China). Hematoxylin and eosin (H&E) kit was purchased from Solarbio (China). Alexa Fluor555-conjugated anti-rabbit IgG (H&L) (#4413) and anti-cleaved-caspase-3 antibody (#9661) were obtained from Cell Signaling Technology (USA). Anti-HSP70 antibody (ab47455), anti-Bax antibody (ab182734), anti-Bcl-2 antibody (ab182858), and anti-pro-paspase-3 antibody (ab32499) were obtained from Abcam (USA). CoraLite488-conjugated beta-actin monoclonal antibody (CL488-66009) was obtained from Proteintech (USA). Fetal bovine serum (FBS), Lyso-Tracker Red DND-99, Mito-Tracker Red FM, and RPMI 1640 medium, were purchased from Thermo Fisher Scientific (USA).

Characterization

The morphologies and elemental distribution were captured on an FEI Tecnai T20 transmission electron microscope. PXRD analysis of various samples was conducted by a Rigaku DMAX-2400 X-ray diffractometer equipped with Cu Kα radiation (λ = 0.154 nm) at 40 kV and 40 mA. X-ray photoelectron spectroscopy (XPS) spectra were carried out using an ESCALAB 250Xi. Fourier transform infrared (FTIR) spectrum was conducted on a Perkin-Elmer 580b spectrophotometer. The dynamic light scattering and zeta potential measurements for different samples were performed on a Malvern Zetasizer Nan Nano ZS90. A UV-1601 spectrophotometer was used for obtaining the

UV−vis absorbance spectra. Electron-spin-resonance (ESR) spectra were recorded by a Bruker EMX1598 spectrometer. The thermal transport properties were measured using LFA 427 Nanoflash (NETZSCH, Germany), and an average reading from three sets of data was obtained. The PA signal intensities were recorded by a ring ultrasound array with an optical parametric oscillator (OPO) (BB-OPO-NIR, Deyang-Tech, Zhejiang, China; pump laser, Nimma-900, Beamtech, Beijing, China) as the light source. The flow cytometry assays were conducted on a BD Accuri C6 flow cytometer (USA). A confocal laser scanning microscope (CLSM, Leica TCS SP8) was used to obtain the fluorescence image. The Cu content was determined *via* inductively coupled plasma optical emission spectrometry (iCAP 6000). The MR images were obtained using a 9.4 T MR scanner (BioSpec 94/20USR).

Photothermal effect of CVS NPs

CVS NPs solutions with gradient concentrations (0, 50, 100, 200, and 400 μg mL^{-1}) were irradiated under 808 nm laser (1.5 W cm^{-2}) for 5 min, and the temperature variations of CVS NPs solution were recorded by an IR thermographic camera. Moreover, the influence of power density was also investigated by recording the temperature of CVS NPs aqueous solution (200 μg mL^{-1}) at varied power densities (0.5, 1.0, 1.5, and 2.0 W cm^{-2}) of the 808 nm laser for 10 min. To further evaluate the photothermal stability of CVS NPs, CVS NPs solution (100 μg mL^{-1}) was irradiated by an 808 nm laser at 1.5 W cm^{-2} during the heating and cooling processes for three cycles. To study the photothermal conversion effect, the CVS NPs aqueous solution (100 μg mL^{-1}) was irradiated under an 808 nm laser (1.5 W cm^{-2}) for 10 min, followed by shutting off the NIR laser. The photothermal conversion efficiency (η) of CVS NPs can be calculated according to the following equation:

$$\eta = \frac{hS(T_{\mathrm{max}} - T_{\mathrm{surr}}) - Q_{\mathrm{dis}}}{I(1 - 10^{A_{808}})} \quad (1)$$

where h (mW m^{-2} °C^{-1}) indicates the thermal conversion efficiency of the system, S (m^2) is the surface area of the container, T_{max} (°C) represents the equilibrium temperature of the sample solution, T_{surr} represents the surrounding ambient temperature, I is the power density of the 808 nm laser (mW), and A_{808} is the absorbance of CVS NP solution at λ = 808 nm. Besides, Q_{dis} represents the heat loss due to the light absorption of the container itself, and it was determined as $Q_{\mathrm{dis}} = (5.4 \times 10^{-4})\ I$ (mW). To calculate hS, another equation was introduced:

$$hS = \frac{mC_{\mathrm{water}}}{\tau_{\mathrm{s}}} \quad (2)$$

where m is the mass of the sample, C_{water} is the heat capacity of water (4.2 J g^{-1} K^{-1}), and the time constant for heat transfer from the system is determined to be τ_s = 559.50 s. Therefore, the hS is calculated to be 7.51 mW °C^{-1}. In addition, the (T_{Max}-T_{Surr}) is 35.1 °C, I_{808} is 1500 mW, and A_{808} is 0.798. By substituting these values into these equations, the 808 nm laser photothermal conversion efficiency (η) of CVS NPs was calculated to be 32.78%.

FDTD simulations

The finite element simulations were performed by using the finite difference-time-domain (FDTD) method with Lumerical FDTD solutions, a software package. The nanostructure is simulated in xy planes with periodic boundary conditions while the broadband plane waves are incident from z directions. Along the propagation directions (z directions), perfect matched layers (PML) are used to absorb all the light coming out to the boundaries. The complex refractive indexes were adopted from the reference (*67*). For structures dispersed in aqueous solutions, the refractive index of the medium (H_2O) was set to 1.33. The geometric parameters for simulations were consistent with the average actual size of the as-prepared samples shown in the TEM image. In the FDTD simulations, the side length of the CVS nanocube was set as 17.4 nm. For the detailed FDTD parameter setting, a unit cell of 300 nm × 300 nm × 300 nm in 3D was employed for the nanostructure. Total-field scattered-field light source with a wavelength range of 400–900 nm was injected into the unit cell along the positive z-axis. The frequency-domain field profile monitor with a fixed wavelength of 808 nm was introduced to acquire field profile data, localized at Z = 0 nm in the x-y plane, Y = 0 nm in the x-z plane, and X = 0 nm in the y-z plane, respectively.

Thermoelectric property measurements

To estimate the thermoelectric properties, the as-prepared powders were first pressed into bulk specimens at 623 K for 15 min under 45 MPa pressure in a vacuum atmosphere, with a heating rate of 100 °C min^{-1}. The resulting cylindrical tablets were sectioned into bars of 11.00 mm × 2.00 mm× 2.00 mm for simultaneous measurements of the temperature-dependent electrical conductivity (σ) and Seebeck coefficient (S) over 300-923 K using a ZEM-3 instrument (Ulvac-Riko ZEM-3). The thermal conductivity (κ) was obtained from $\kappa = D \times C_P \times d$, where D, C_P, and d mean the thermal diffusivity, heat capacity, and mass density, respectively. The thermal diffusivity (D) was detected using an LFA 457 (NETZSCH) under high-purity helium atmosphere. The heat

capacity (C_P) was estimated by using the Dulong-Petit rule. The Archimedes method was performed to calculate the density (d).

Cell culture

The 4T1 murine mammary carcinoma cell line was acquired from FDCC (Ruilu in Shanghai, China) and cultured in RPMI 1640 medium with 10% FBS and 1% Penicillin-Streptomycin. Raw 264.7 murine mononuclear macrophage cell line was obtained from the American Type Culture Collection (ATCC) and cultured in Dulbecco's Modified Eagle Medium supplemented with 10% FBS and 1% Penicillin-Streptomycin. The cell lines were incubated in the incubator. All the cultures were conducted in the incubator at 37 °C and 5% CO_2 atmosphere.

***In vivo* biodistribution of CVS NPs**

Female BALB/c mice (n = 3) were i.v. injected with CVS NPs (100 μL, 15 mg kg^{-1}). At 1, 3, 6, 12, 24, and 48 h post-injection, these mice were sacrificed to collect their major organs including liver, spleen, kidney, heart, lung, and tumor for biodistribution analysis *via* an inductively coupled plasma−optical emission spectrometry (ICP-OES). The biodistributions of CVS NPs in different organs and tumors were calculated as the Cu percentage of the injected dose per gram of tissue.

Histochemical analysis

At the end of therapy, tumor tissues of mice in each treatment group were collected followed by fixed in 37% formalin and embedded in paraffin for one day. Then, paraffin-embedded tumor sections (4 μm) were cut by a microtome (Leica RM2235, Germany). For the pathological investigation, the obtained samples were stained with hematoxylin and eosin (H&E) and TdT-mediated dUTP nick-end labeling (TUNEL) by following the standard protocol, and the slides were visualized using a CLSM. For immunofluorescence staining, the sections were placed into xylene two times for 15 min to remove paraffin, hydrated through an ethanol gradient (100%, 95%, 90%, 80%, and 70%), and deionized water for 5 min in each solution. Then, the antigen retrieval was conducted by sodium citrate buffer solution (0.01 M) at 95 °C for 15 min. Subsequently, the obtained samples were incubated with primary antibodies of Anti-HSP70 antibody (1:200 dilution), at 4 °C overnight, followed by incubation with Alexa Fluor488-conjugated anti-rabbit IgG antibody (1:1000 dilution) for 1.5 h at 37 °C. After washing with PBS for three times, the samples were

stained with DAPI solution for 5 min at room temperature. At last, the stained tissues were photographed by using a CLSM.

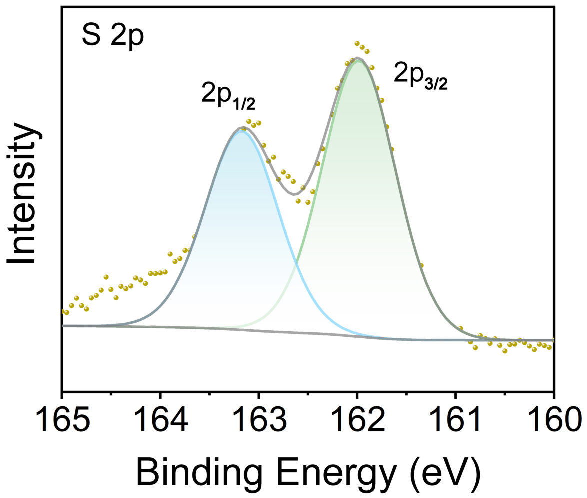

Fig. S1. S2p core-level XPS spectrum.

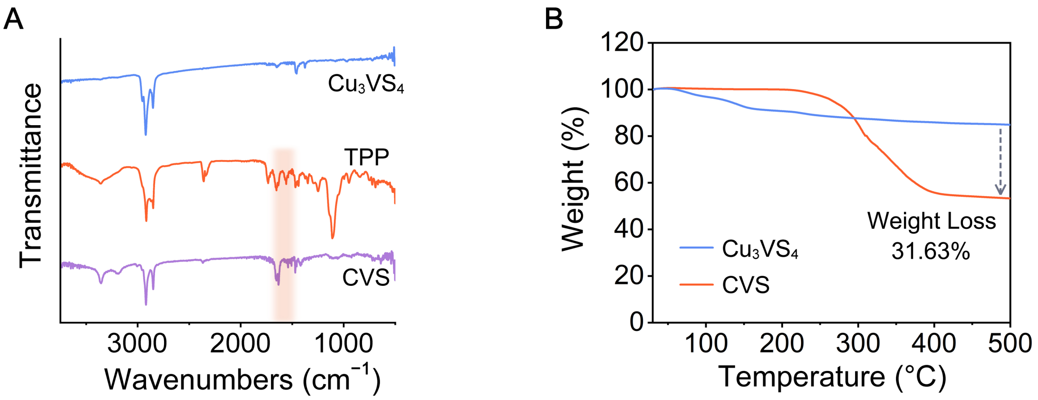

Fig. S2. FT-IR spectra and TGA curves. (**A**) FT-IR spectra of Cu_3VS_4, TPP, and CVS. Compared to the Cu_3VS_4 sample, the additional peaks of benzene from 1600 to 1400 cm^{-1} in the CVS sample indicate the successful modification of DSPE-PEG-TPP. (**B**) Thermogravimetric analysis (TGA) curves of Cu_3VS_4 and CVS.

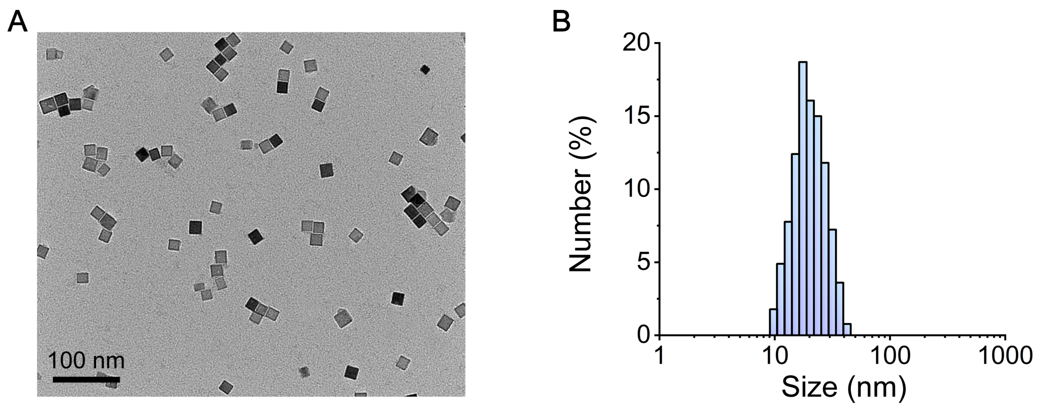

Fig. S3. TEM image and size distribution. (**A**) TEM image and (**B**) particle-size distribution of CVS NPs.

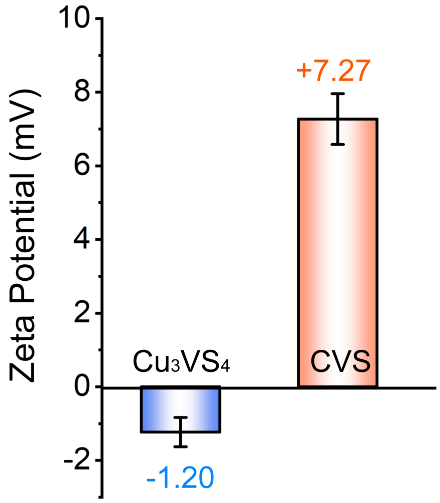

Fig. S4. Zeta potential values of Cu_3VS_4 and CVS nanoparticles, respectively. Data are represented as mean ± S.D. (n = 3 represents three independent samples).

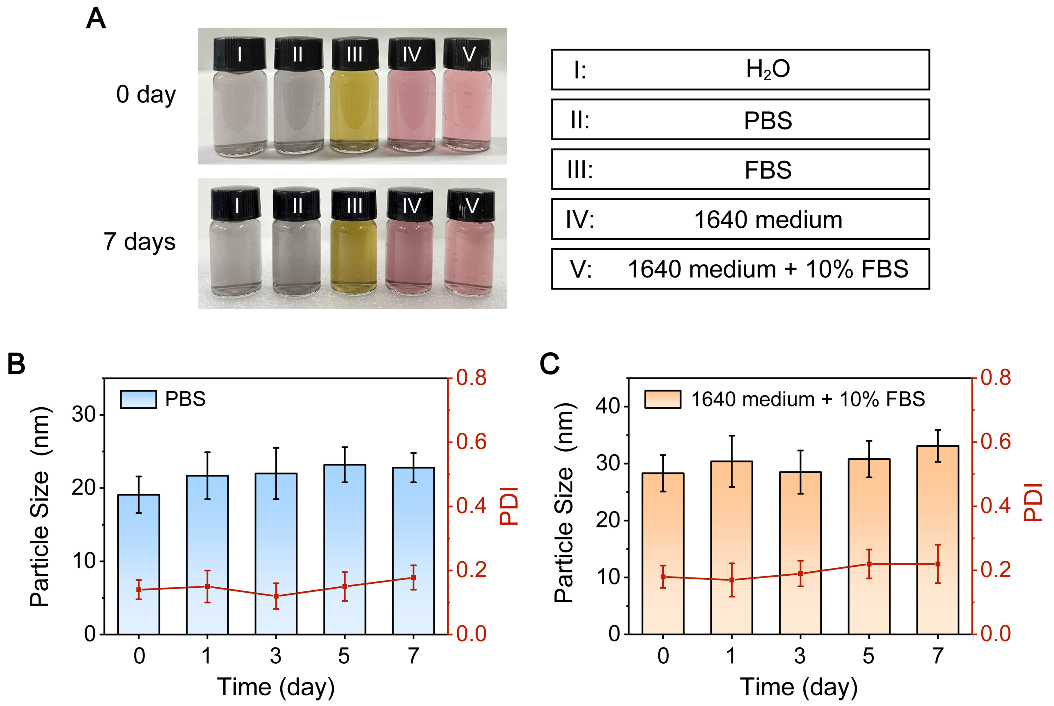

Fig. S5. Dispersibility and nanoparticle size. (**A**) Dispersibility of CVS NPs (100 μg mL^{-1}) in different physiological media. The average nanoparticle size and the corresponding PDI of CVS NPs dispersed in (**B**) PBS and (**C**) RPMI 1640 medium containing 10% FBS within 7 days. Data are displayed as mean ± S.D. (n = 3).

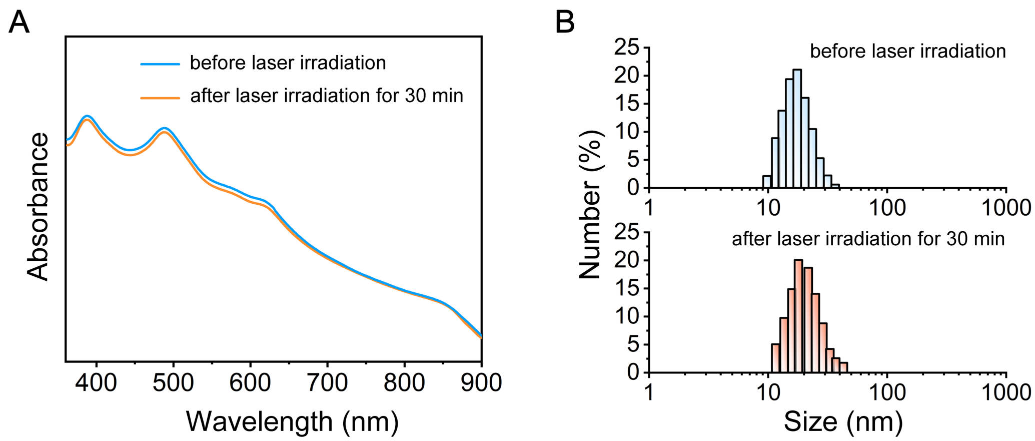

Fig. S6. UV-vis spectra and size distribution. (**A**) UV-vis absorbance spectra and (**B**) particle-size distributions of CVS NPs before and after laser radiation for 30 min.

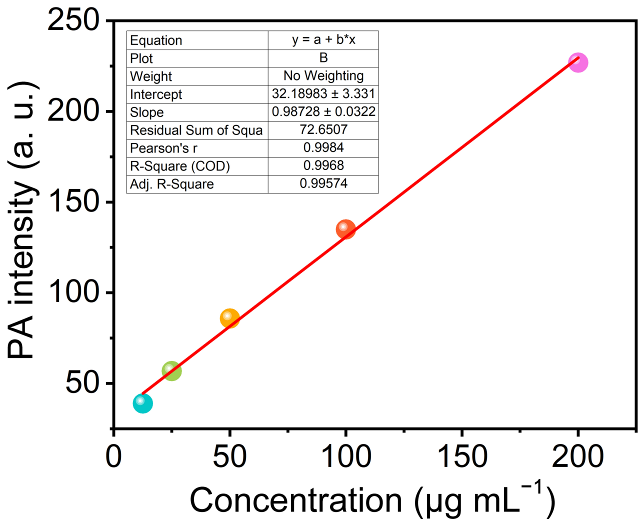

Fig. S7. Linear fit of PA intensity of CVS NPs as a function of sample concentration at 808 nm.

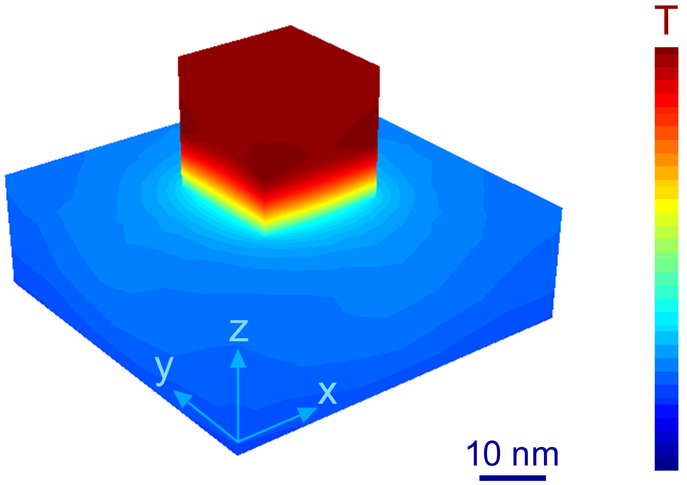

Fig. S8. Steady-state temperature (T) of the Cu_3VS_4 nanostructure under 808 nm laser irradiation as calculated by FDTD simulation.

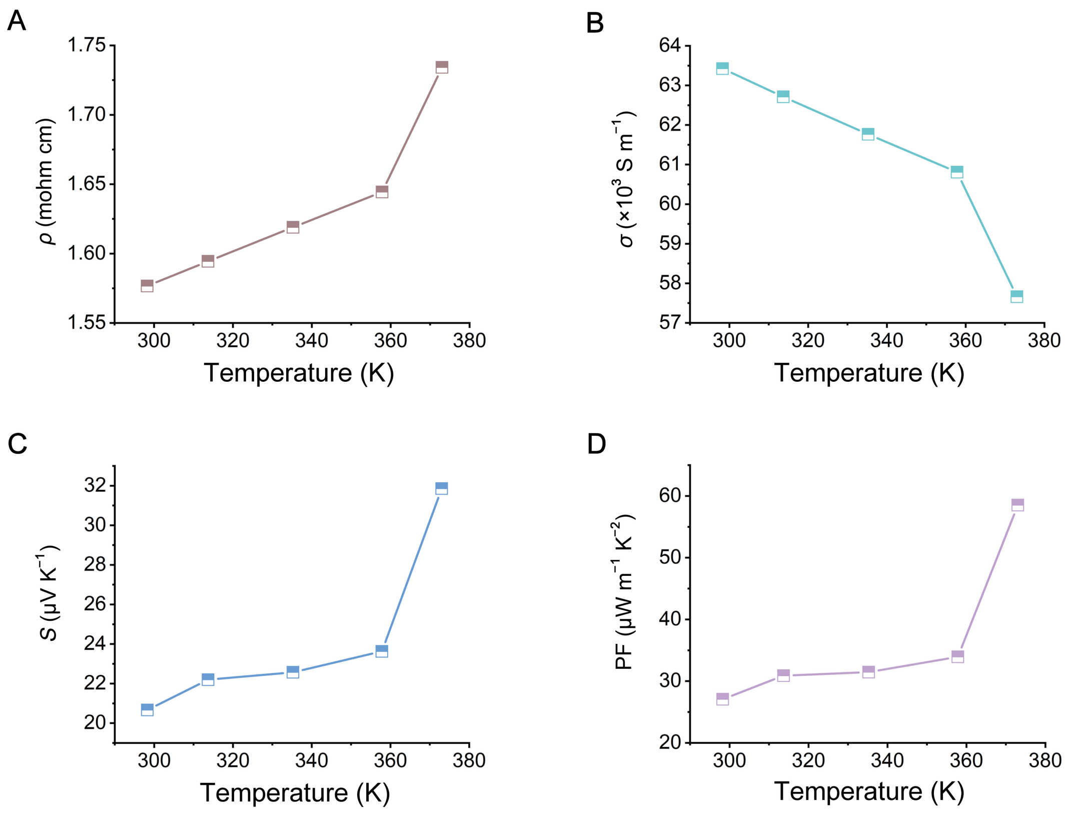

Fig. S9. Electrical properties of Cu_3VS_4. (**A**) Electrical resistivity ρ, (**B**) electrical conductivity σ, (**C**) Seebeck coefficient, and (**D**) power factor for Cu_3VS_4.

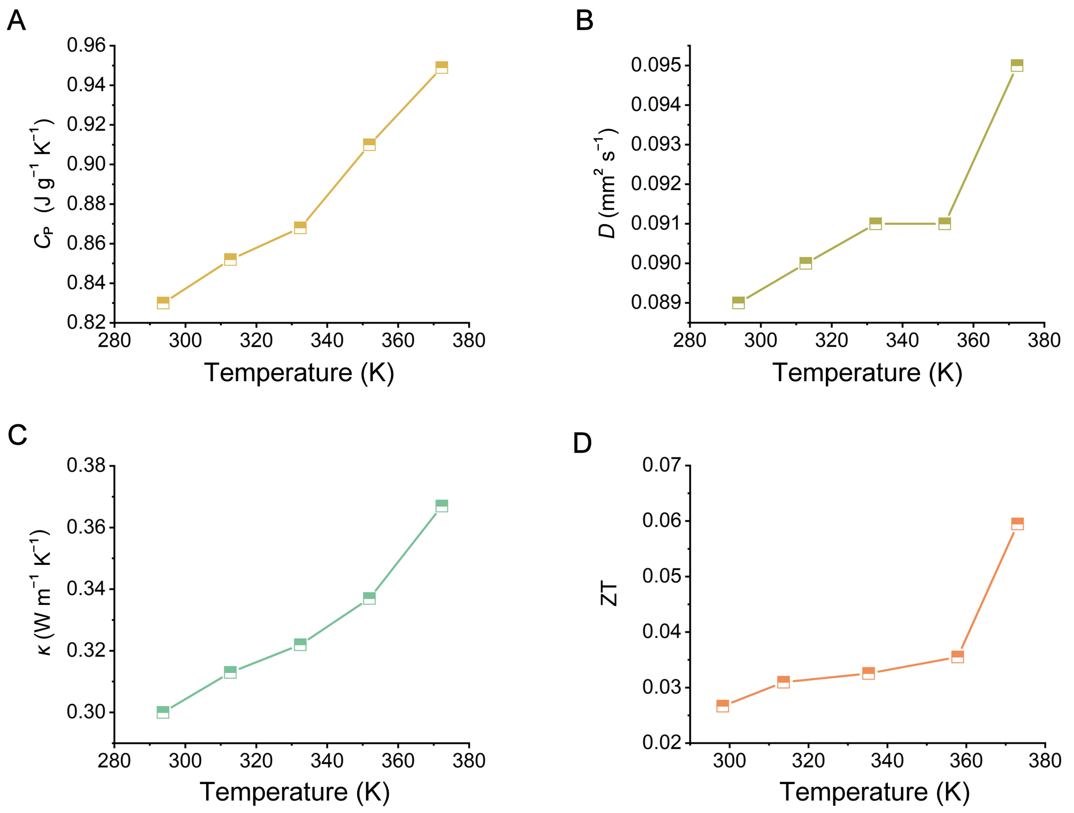

Fig. S10. Temperature-dependent thermoelectric properties of Cu_3VS_4. (**A**) Specific heat C_P, (**B**) thermal diffusivity D, (**C**) thermal conductivity κ, together with (**D**) dimensionless figure of merit ZT.

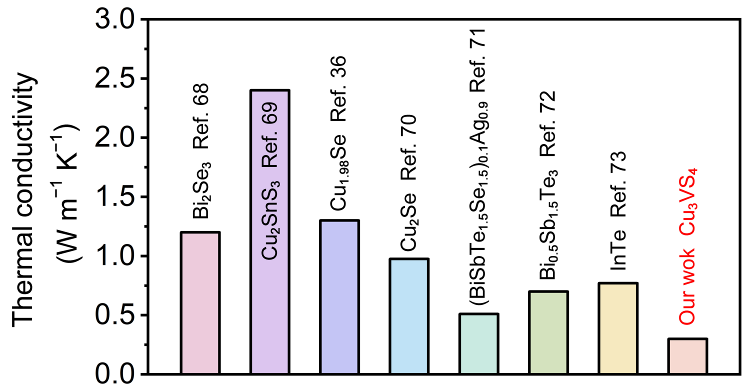

Fig. S11. Comparison on the thermal conductivity of Cu_3VS_4 with some typical inorganic thermoelectric materials at room temperature (*36*, *68-73*).

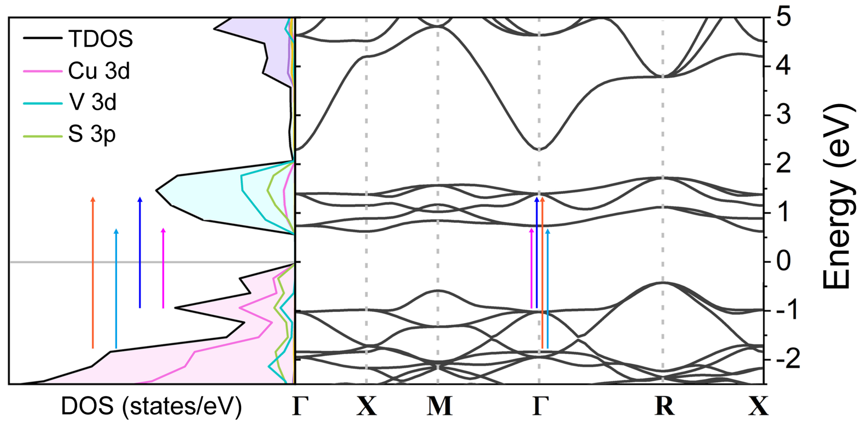

Fig. S12. Band structure of Cu_3VS_4 with the optical transitions for absorption peak.

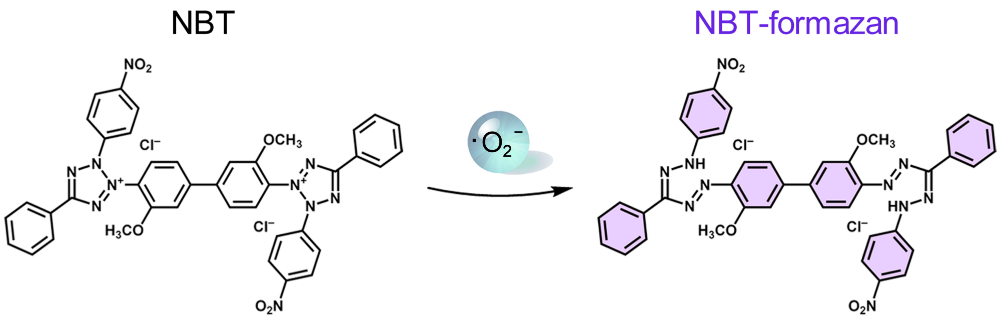

Fig. S13. Schematic illustration for the redox reaction of NBT with $\cdot O_2^-$, which can be used for monitoring intracellular $\cdot O_2^-$ content.

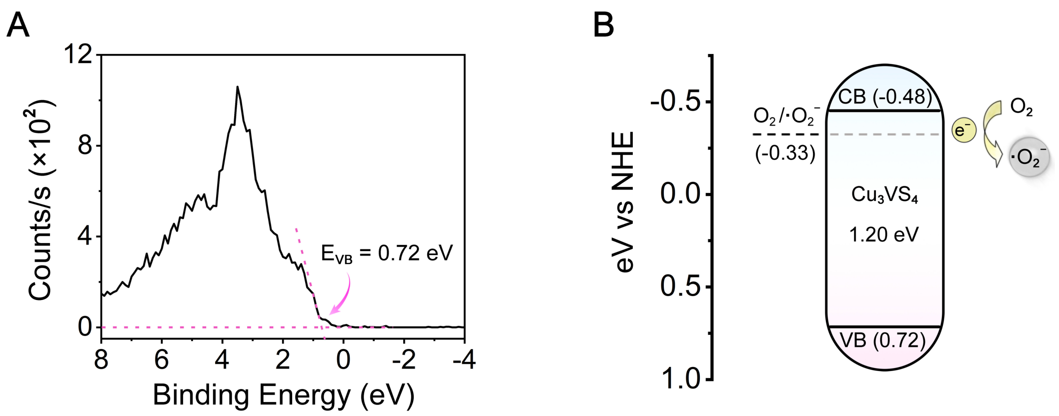

Fig. S14. Valence band XPS spectrum and schematic illustration. (**A**) Valence band XPS spectrum of Cu_3VS_4 NPs, representing the valence band maximum position. (**B**) Schematic illustration of the energy bands of Cu_3VS_4 NPs, showing that the formation of $\cdot O_2^-$ free radical is possible.

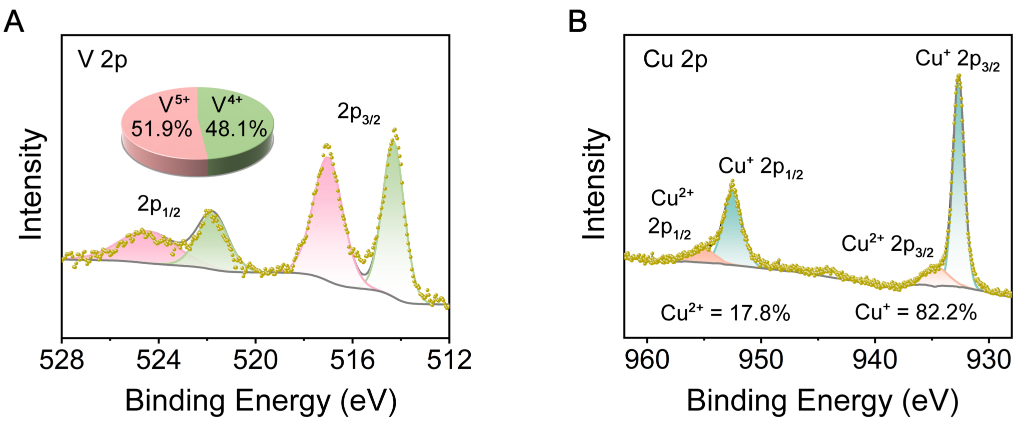

Fig. S15. XPS spectra. (**A**) High-resolution V 2p and (**B**) Cu 2p XPS spectra of CVS NPs after H_2O_2 treatment for 12 h. The peaks at 935.1 and 954.3 eV correspond to the electron energy peaks of Cu^{2+} $2p_{3/2}$ and $2p_{1/2}$, respectively. Two valence states (Cu^{2+}/Cu^{+}) were simultaneously presented in the CVS NPs after reacting with H_2O_2. On the basis of these experimental findings, it was proposed that the CVS NPs could respond specifically to H_2O_2 through the redox reaction between Cu^{+}/V^{4+} in the CVS NPs and H_2O_2, according to the following reaction equations: $H^{+} + Cu^{+} + H_2O_2 \rightarrow Cu^{2+} + \cdot OH + H_2O$, and $H^{+} + V^{4+} + H_2O_2 \rightarrow V^{5+} + \cdot OH + H_2O$.

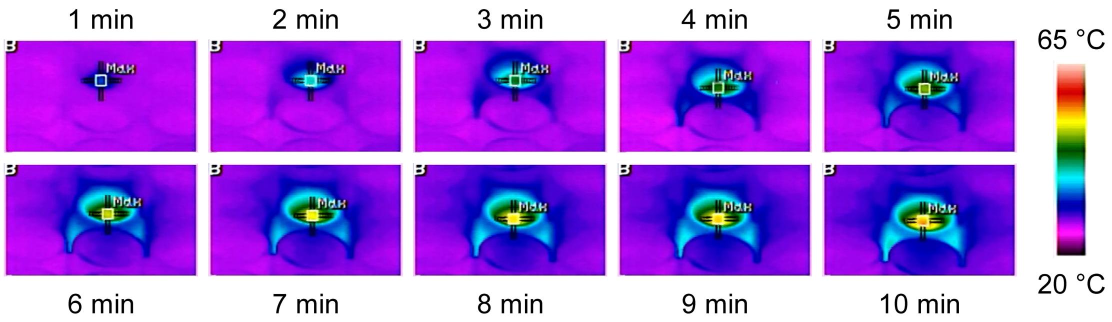

Fig. S16. *In vivo* infrared thermal images of the 4T1 cells incubated with CVS NPs (100 μg mL^{-1}) irradiated by an 808 nm laser (1.0 W cm^{-2}).

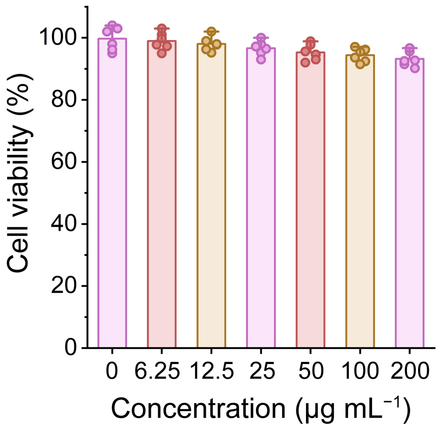

Fig. S17. Cell viability of RAW 264.7 cells after incubation with CVS NPs for 24 h.

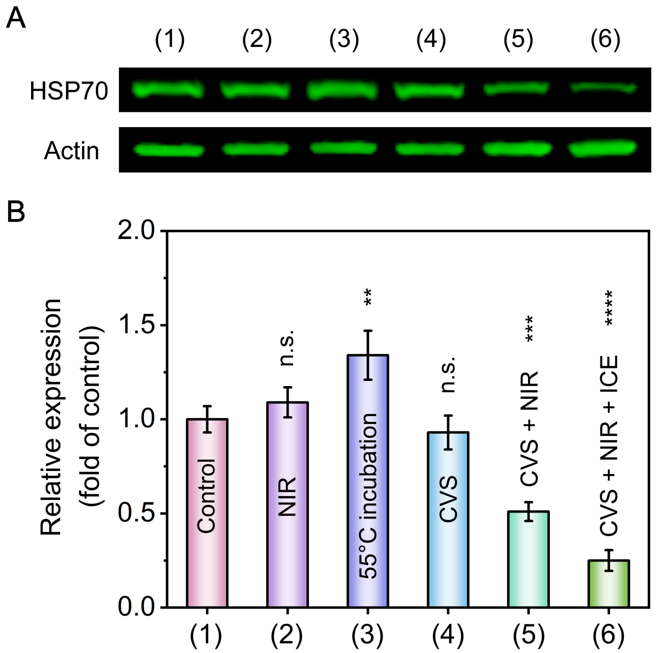

Fig. S18. Western blot analysis and quantitative analysis. (**A**) Western blot analysis and (**B**) quantitative analysis of HSP70 expression levels in 4T1 cells with different treatments. Statistical analysis was performed *via* one-way ANOVA with Tukey's multiple comparisons post hoc test. *P < 0.05; **P < 0.01; ***P < 0.001; ****P < 0.0001; n.s., not significance. Data were presented as mean ± S.D. (n = 3).

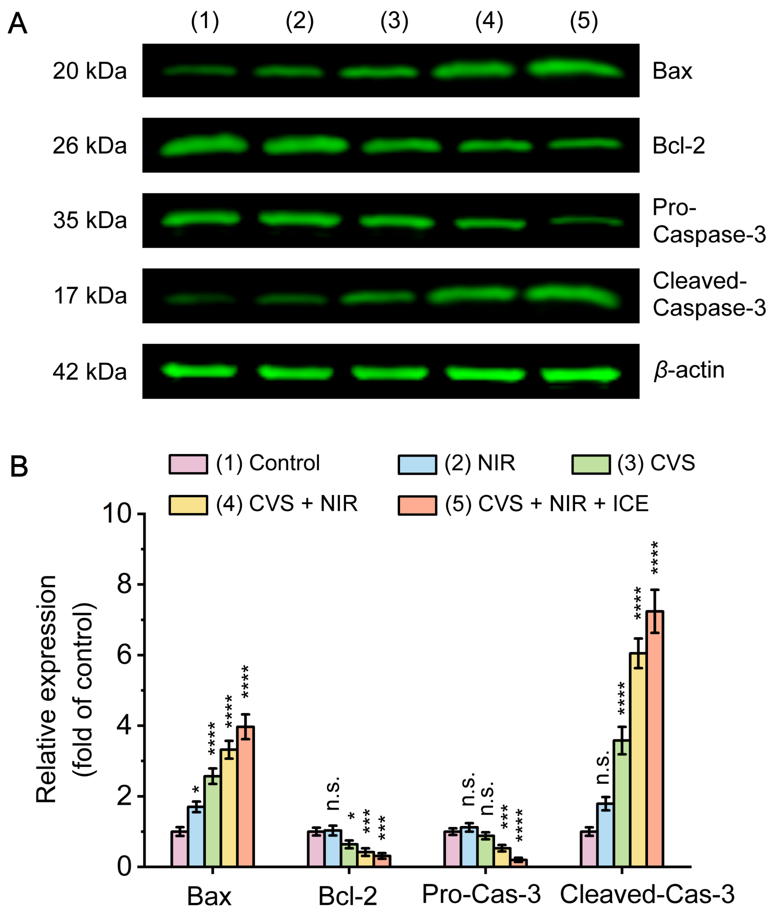

Fig. S19. Western blot analysis and quantitative analysis. (**A**) Western blot analysis and (**B**) quantitative analysis of Bax, Bcl-2, pro-caspase-3, and cleaved-caspase-3 protein expression in 4T1 cells after different treatments. Statistical analysis was performed *via* one-way ANOVA with Tukey's multiple comparisons post hoc test. *P < 0.05; **P < 0.01; ***P < 0.001; ****P < 0.0001; n.s., not significance. Data were presented as mean ± S.D. (n = 3).

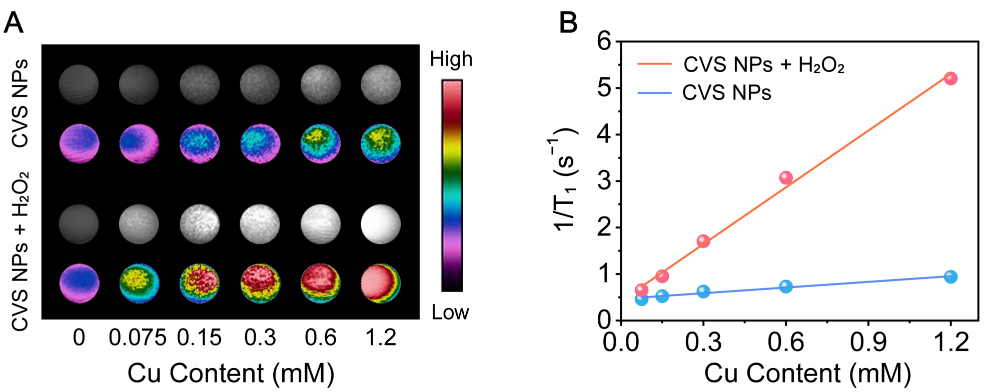

Fig. S20. *In vitro* T_1-MR images and relaxation rate r_1 *versus* Cu concentration. (**A**) *In vitro* T_1-MR images of CVS NPs with different concentrations (obtained under 9.4-T magnetic resonance scanner). (**B**) Relaxation rate r_1 *versus* Cu concentration of CVS NPs with or without the addition of H_2O_2.

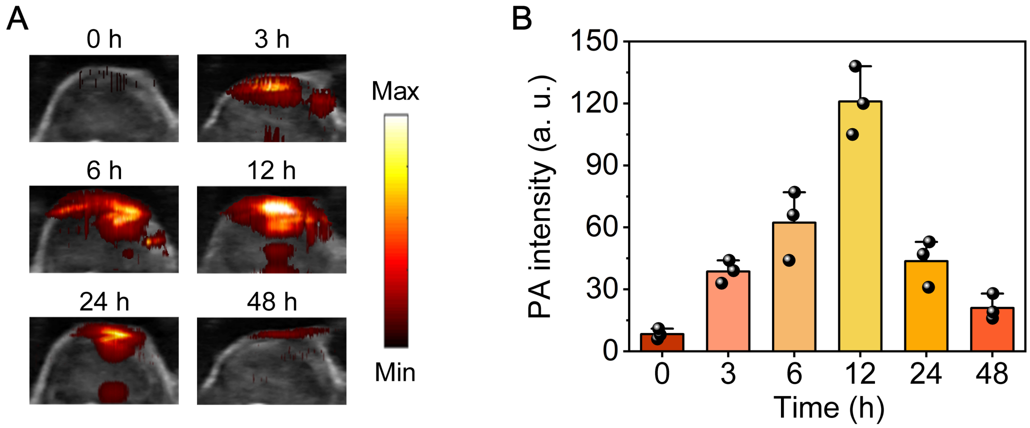

Fig. S21. PA images and quantification of PA intensities. (**A**) PA images of living mice bearing xenograft 4T1 tumors at 0, 3, 6, 12, 24, and 48 h after i.v. injection of CVS NPs. The PA images were acquired at 808 nm. (**B**) Quantification of PA intensities at the tumor regions after various durations. (n = 3).

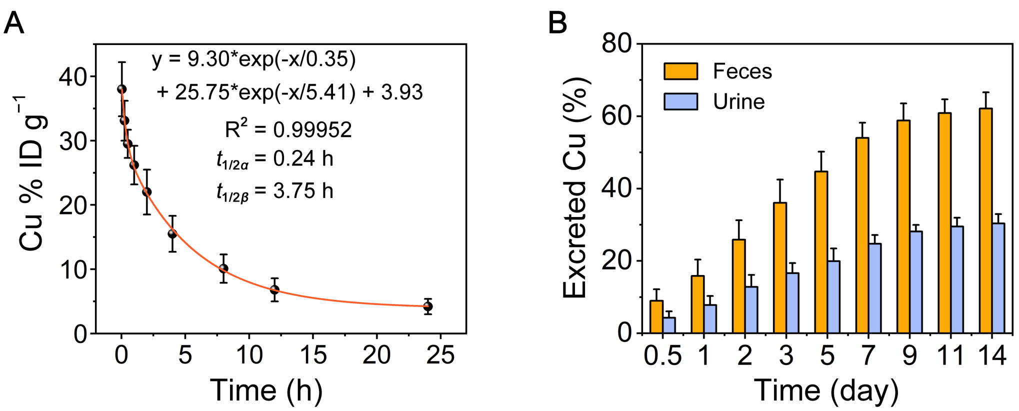

Fig. S22. Blood circulation curve and accumulated Cu amount. (**A**) Blood circulation curve of intravenously injected CVS NPs. Data are expressed as mean ± S.D. (n = 3). (**B**) Accumulated Cu in feces and urine excreted out of the mouse body within 14 days after i.v. injection of CVS NPs (n = 3).

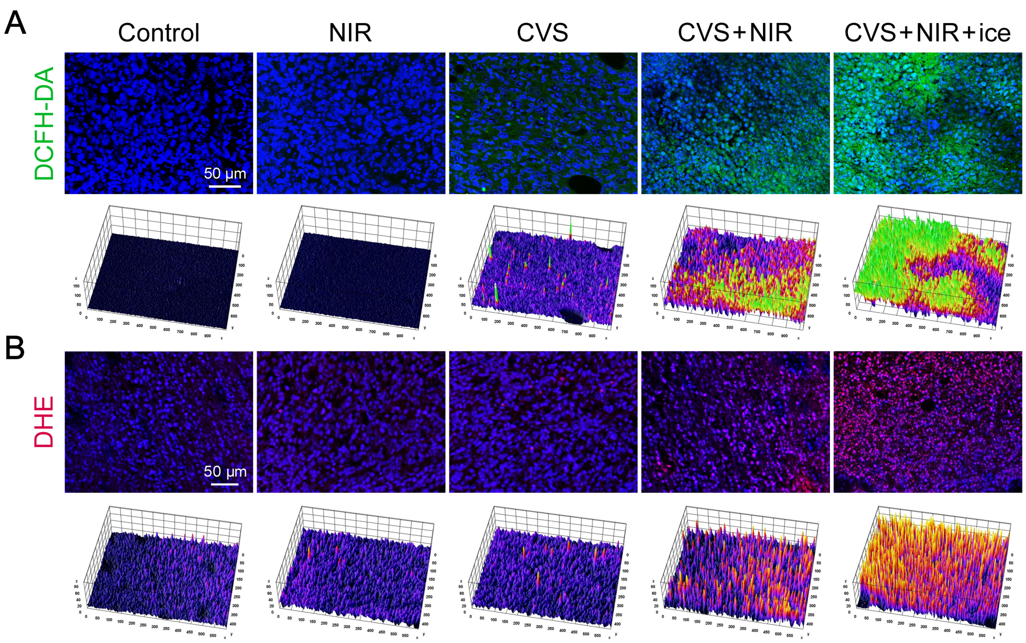

Fig. S23. DCFH-DA and DHE staining images. (**A**) DCFH-DA and (**B**) DHE staining images of tumor slices obtained from mice receiving different treatments.

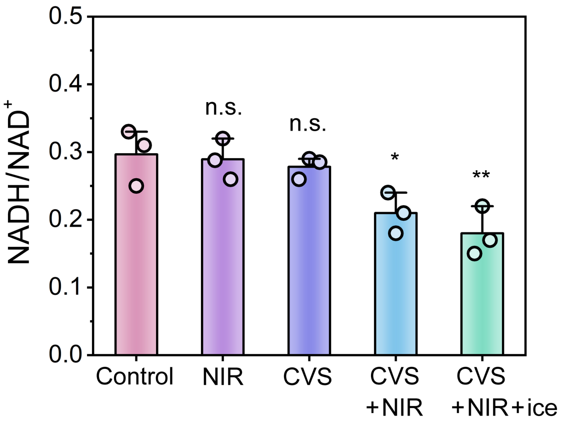

Fig. S24. NADH/NAD$^+$ redox ratios in the tumor regions of mice after different treatments. Statistical analysis was performed *via* one-way ANOVA with Tukey's multiple comparisons post hoc test. *P < 0.05; **P < 0.01; n.s., not significance. Data were represented as mean ± S.D. from three independent replicates.

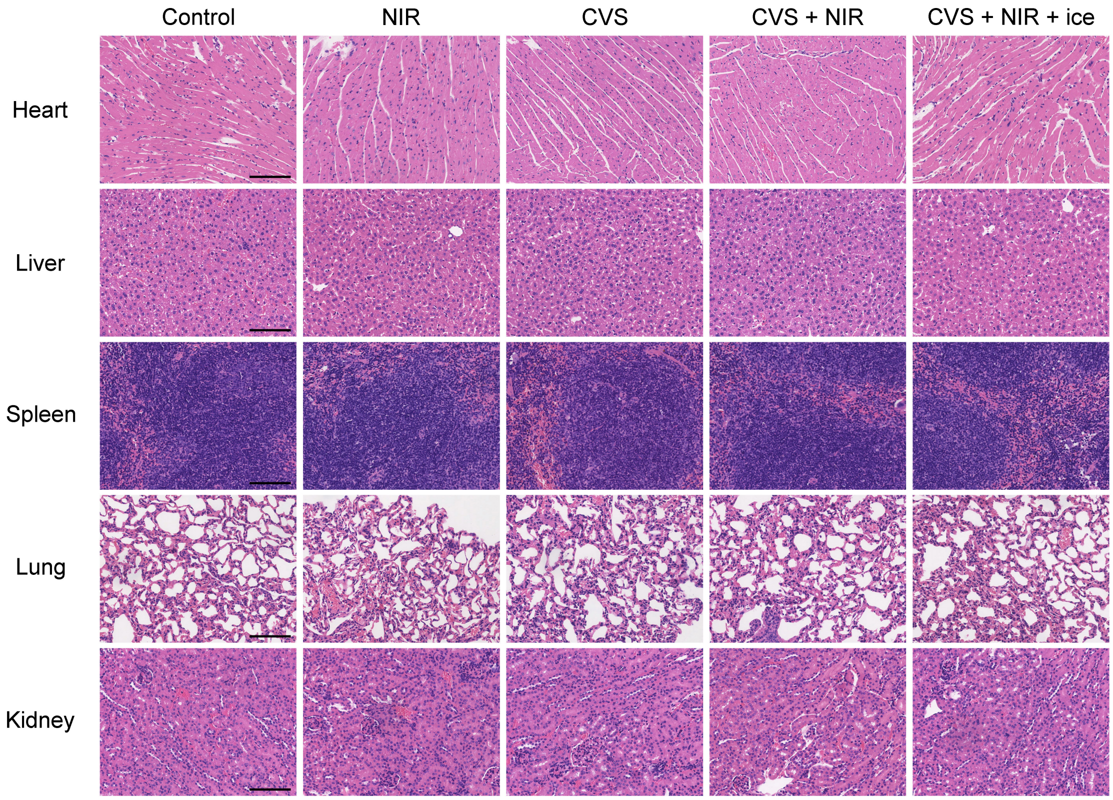

Fig. S25. H&E staining of normal tissues (heart, liver, spleen, lung, and kidney) obtained from different treatment groups. Scale bar: 100 μm.

Legend for movie S1

Movie S1. A representative video showing the electric field enhancement is localized around the nanostructure.

REFERENCES AND NOTES

1. J. Nam, S. Son, K. S. Park, W. Zou, L. D. Shea, J. J. Moon, Cancer nanomedicine for combination cancer immunotherapy. *Nat. Rev. Mater.* **4**, 398–414 (2019).

2. X. Li, J. F. Lovell, J. Yoon, X. Chen, Clinical development and potential of photothermal and photodynamic therapies for cancer. *Nat. Rev. Clin. Oncol.* **17**, 657–674 (2020).

3. L. Cheng, C. Wang, L. Feng, K. Yang, Z. Liu, Functional nanomaterials for phototherapies of cancer. *Chem. Rev.* **114**, 10869–10939 (2014).

4. H. Wen, K. Tamarov, E. Happonen, V.-P. Lehto, W. Xu, Inorganic nanomaterials for photothermal-based cancer theranostics. *Adv. Ther.* **4**, 2000207 (2021).

5. E. W. Gerner, M. J. Schneider, Induced thermal resistance in HeLa cells. *Nature* **256**, 500–502 (1975).

6. H. M. Beere, 'The stress of dying': The role of heat shock proteins in the regulation of apoptosis. *J. Cell Sci.* **117**, 2641–2651 (2004).

7. G. Jego, A. Hazoumé, R. Seigneuric, C. Garrido, Targeting heat shock proteins in cancer. *Cancer Lett.* **332**, 275–285 (2013).

8. Y. Yang, W. Zhu, Z. Dong, Y. Chao, L. Xu, M. Chen, Z. Liu, 1D coordination polymer nanofibers for low-temperature photothermal therapy. *Adv. Mater.* **29**, 1703588 (2017).

9. G. Gao, Y.-W. Jiang, W. Sun, Y. Guo, H.-R. Jia, X.-W. Yu, G.-Y. Pan, F.-G. Wu, Molecular targeting-mediated mild-temperature photothermal therapy with a smart albumin-based nanodrug. *Small* **15**, e1900501 (2019).

10. X. Deng, W. Guan, X. Qing, W. Yang, Y. Que, L. Tan, H. Liang, Z. Zhang, B. Wang, X. Liu, Y. Zhao, Z. Shao, Ultrafast low-temperature photothermal therapy activates autophagy and recovers immunity for efficient antitumor treatment. *ACS Appl. Mater. Interfaces* **12**, 4265–4275 (2020).

11. M. Taipale, D. F. Jarosz, S. Lindquist, HSP90 at the hub of protein homeostasis: emerging mechanistic insights. *Nat. Rev. Mol. Cell Biol.* **11**, 515–528 (2010).

12. J. Zhou, M. Li, Y. Hou, Z. Luo, Q. Chen, H. Cao, R. Huo, C. Xue, L. Sutrisno, L. Hao, Y. Cao, H. Ran, L. Lu, K. Li, K. Cai, Engineering of a nanosized biocatalyst for combined tumor starvation and low-temperature photothermal therapy. *ACS Nano* **12**, 2858–2872 (2018).

13. M. Chang, Z. Hou, M. Wang, D. Wen, C. Li, Y. Liu, Y. Zhao, J. Lin, Cu single atom nanozyme based high-efficiency mild photothermal therapy through cellular metabolic regulation. *Angew. Chem. Int. Ed.* **61**, e202209245 (2022).

14. E. L. Pearce, M. C. Poffenberger, C. H. Chang, R. G. Jones, Fueling immunity: Insights into metabolism and lymphocyte function. *Science* **342**, 1242454 (2013).

15. C. H. Chang, J. Qiu, D. O'Sullivan, M. D. Buck, T. Noguchi, J. D. Curtis, Q. Chen, M. Gindin, M. M. Gubin, G. J. W. van der Windt, E. Tonc, R. D. Schreiber, E. J. Pearce, E. L. Pearce, Metabolic competition in the tumor microenvironment is a driver of cancer progression. *Cell* **162**, 1229–1241 (2015).

16. J. B. Spinelli, M. C. Haigis, The multifaceted contributions of mitochondria to cellular metabolism. *Nat. Cell Biol.* **20**, 745–754 (2018).

17. L. Cui, A. M. Gouw, E. L. LaGory, S. Guo, N. Attarwala, Y. Tang, J. Qi, Y.-S. Chen, Z. Gao, K. M. Casey, A. A. Bazhin, M. Chen, L. Hu, J. Xie, M. Fang, C. Zhang, Q. Zhu, Z. Wang, A. J. Giaccia, S. S. Gambhir, W. Zhu, D. W. Felsher, M. D. Pegram, E. A. Goun, A. Le, J. Rao, Mitochondrial copper depletion suppresses triple-negative breast cancer in mice. *Nat. Biotechnol.* **39**, 357–367 (2021).

18. L. A. Sazanov, A giant molecular proton pump: structure and mechanism of respiratory complex I. *Nat. Rev. Mol. Cell Biol.* **16**, 375–388 (2015).

19. J. Gu, T. Liu, R. Guo, L. Zhang, M. Yang, The coupling mechanism of mammalian mitochondrial complex I. *Nat. Struct. Mol. Biol.* **29**, 172–182 (2022).

20. X. Lu, L. Sun, P. Jiang, X. Bao, Progress of photodetectors based on the photothermoelectric effect. *Adv. Mater.* **31**, e1902044 (2019).

21. X. Lu, P. Jiang, X. Bao, Phonon-enhanced photothermoelectric effect in $SrTiO_3$ ultra-broadband photodetector. *Nat. Commun.* **10**, 138 (2019).

22. B. Lv, Y. Liu, W. Wu, Y. Xie, J.-L. Zhu, Y. Cao, W. Ma, N. Yang, W. Chu, Y. Jia, J. Wei, J.-L. Sun, Local large temperature difference and ultra-wideband photothermoelectric response of the silver nanostructure film/carbon nanotube film heterostructure. *Nat. Commun.* **13**, 1835 (2022).

23. R. Wang, Z. He, J.-L. Wang, J.-Y. Liu, J.-W. Liu, S.-H. Yu, Manipulating nanowire structures for an enhanced broad-band flexible photothermoelectric photodetector. *Nano Lett.* **22**, 5929–5935 (2022).

24. L. E. Bell, Cooling, heating, generating power, and recovering waste heat with thermoelectric systems. *Science* **321**, 1457–1461 (2008).

25. L.-D. Zhao, S.-H. Lo, Y. Zhang, H. Sun, G. Tan, C. Uher, C. Wolverton, V. P. Dravid, M. G. Kanatzidis, Ultralow thermal conductivity and high thermoelectric figure of merit in SnSe crystals. *Nature* **508**, 373–377 (2014).

26. T. Zhu, Y. Liu, C. Fu, J. P. Heremans, J. G. Snyder, X. Zhao, Compromise and synergy in high-efficiency thermoelectric materials. *Adv. Mater.* **29**, 1605884 (2017).

27. X.-L. Shi, J. Zou, Z.-G. Chen, Advanced thermoelectric design: From materials and structures to devices. *Chem. Rev.* **120**, 7399–7515 (2020).

28. N. M. Gabor, J. C. W. Song, Q. Ma, N. L. Nair, T. Taychatanapat, K. Watanabe, T. Taniguchi, L. S. Levitov, P. Jarillo-Herrero, Hot carrier-assisted intrinsic photoresponse in graphene. *Science* **334**, 648–652 (2011).

29. D. Wu, K. Yan, Y. Zhou, H. Wang, L. Lin, H. Peng, Z. Liu, Plasmon-enhanced photothermoelectric conversion in chemical vapor deposited graphene p–n junctions. *J. Am. Chem. Soc.* **135**, 10926–10929 (2013).

30. X. Cai, A. B. Sushkov, R. J. Suess, M. M. Jadidi, G. S. Jenkins, L. O. Nyakiti, R. L. Myers-Ward, S. Li, J. Yan, D. K. Gaskill, T. E. Murphy, H. D. Drew, M. S. Fuhrer, Sensitive room-temperature terahertz detection *via* the photothermoelectric effect in graphene. *Nat. Nanotechnol.* **9**, 814–819 (2014).

31. M. Buscema, M. Barkelid, V. Zwiller, H. S. J. van der Zant, G. A. Steele, A. Castellanos-Gomez, Large and tunable photothermoelectric effect in single-layer MoS_2. *Nano Lett.* **13**, 358–363 (2013).

32. K. W. Mauser, S. Kim, S. Mitrovic, D. Fleischman, R. Pala, K. C. Schwab, H. A. Atwater, Resonant thermoelectric nanophotonics. *Nat. Nanotechnol.* **12**, 770–775 (2017).

33. G. Dennler, R. Chmielowski, S. Jacob, F. Capet, P. Roussel, S. Zastrow, K. Nielsch, I. Opahle, G. K. H. Madsen, Are binary copper sulfides/selenides really new and promising thermoelectric materials? *Adv. Energy Mater.* **4**, 1301581 (2014).

34. P. Qiu, X. Shi, L. Chen, Cu-based thermoelectric materials. *Energy Storage Mater.* **3**, 85–97 (2016).

35. P. Lemoine, G. Guélou, B. Raveau, E. Guilmeau, Crystal structure classification of copper-based sulfides as a tool for the design of inorganic functional materials. *Angew. Chem. Int. Ed.* **61**, e202108686 (2022).

36. H. Liu, X. Shi, F. Xu, L. Zhang, W. Zhang, L. Chen, Q. Li, C. Uher, T. Day, G. J. Snyder, Copper ion liquid-like thermoelectrics. *Nat. Mater.* **11**, 422–425 (2012).

37. K. S. Weldert, W. G. Zeier, T. W. Day, M. Panthöfer, G. J. Snyder, W. Tremel, Thermoelectric transport in Cu_7PSe_6 with high copper ionic mobility. *J. Am. Chem. Soc.* **136**, 12035–12040 (2014).

38. P. Qiu, M. T. Agne, Y. Liu, Y. Zhu, H. Chen, T. Mao, J. Yang, W. Zhang, S. M. Haile, W. G. Zeier, J. Janek, C. Uher, X. Shi, L. Chen, G. J. Snyder, Suppression of atom motion and metal deposition in mixed ionic electronic conductors. *Nat. Commun.* **9**, 2910 (2018).

39. K. Zhao, P. Qiu, X. Shi, L. Chen, Recent advances in liquid-like thermoelectric materials. *Adv. Funct. Mater.* **30**, 1903867 (2020).

40. Y. He, T. Day, T. Zhang, H. Liu, X. Shi, L. Chen, G. J. Snyder, High thermoelectric performance in non-toxic earth-abundant copper sulfide. *Adv. Mater.* **26**, 3974–3978 (2014).

41. L. Zhao, X. Wang, F. Y. Fei, J. Wang, Z. Cheng, S. Dou, J. Wang, G. J. Snyder, High thermoelectric and mechanical performance in highly dense $Cu_{2-x}S$ bulks prepared by a melt-solidification technique. *J. Mater. Chem. A* **3**, 9432–9437 (2015).

42. J.-Y. Tak, W. H. Nam, C. Lee, S. Kim, Y. S. Lim, K. Ko, S. Lee, W.-S. Seo, H. K. Cho, J.-H. Shim, C.-H. Park, Ultralow lattice thermal conductivity and significantly enhanced near-room-temperature thermoelectric figure of merit in α-Cu_2Se through suppressed Cu vacancy formation by overstoichiometric Cu addition. *Chem. Mater.* **30**, 3276–3284 (2018).

43. V. Mantella, S. Ninova, S. Saris, A. Loiudice, U. Aschauer, R. Buonsanti, Synthesis and size-dependent optical properties of intermediate band gap Cu_3VS_4 nanocrystals. *Chem. Mater.* **31**, 532–540 (2019).

44. V. Mantella, S. B. Varandili, J. R. Pankhurst, R. Buonsanti, Colloidal synthesis of Cu–M–S (M = V, Cr, Mn) nanocrystals by tuning the copper precursor reactivity. *Chem. Mater.* **32**, 9780–9786 (2020).

45. D. Petritis, G. Martinez, C. Levy-Clement, O. Gorochov, Investigation of the vibronic properties of Cu_3VS_4, Cu_3NbS_4, and Cu_3TaS_4 compounds. *Phys. Rev. B* **23**, 6773–6786 (1981).

46. S. Ghosh, T. Avellini, A. Petrelli, I. Kriegel, R. Gaspari, G. Almeida, G. Bertoni, A. Cavalli, F. Scotognella, T. Pellegrino, L. Manna, Colloidal $CuFeS_2$ nanocrystals: Intermediate Fe d-band leads to high photothermal conversion efficiency. *Chem. Mater.* **28**, 4848–4858 (2016).

47. X. Jiang, S. Zhang, F. Ren, L. Chen, J. Zeng, M. Zhu, Z. Cheng, M. Gao, Z. Li, Ultrasmall magnetic $CuFeSe_2$ ternary nanocrystals for multimodal imaging guided photothermal therapy of cancer. *ACS Nano* **11**, 5633–5645 (2017).

48. B. Li, F. Yuan, G. He, X. Han, X. Wang, J. Qin, Z. X. Guo, X. Lu, Q. Wang, I. P. Parkin, C. Wu, Ultrasmall $CuCo_2S_4$ nanocrystals: All-in-one theragnosis nanoplatform with magnetic resonance/near-infrared imaging for efficiently photothermal therapy of tumors. *Adv. Funct. Mater.* **27**, 1606218 (2017).

49. J. He, T. M. Tritt, Advances in thermoelectric materials research: Looking back and moving forward. *Science* **357**, eaak9997 (2017).

50. Y. Zhang, X. Yuan, X. Sun, B.-C. Shih, P. Zhang, W. Zhang, Comparative study of structural and electronic properties of Cu-based multinary semiconductors. *Phys. Rev. B* **84**, 075127 (2011).

51. J. Shen, H. Yu, Y. Pei, Y. Chen, Resonant doping in BiCuSeO thermoelectrics from first principles. *J. Mater. Chem. A* **5**, 931–936 (2017).

52. Y. Pei, X. Shi, A. LaLonde, H. Wang, L. Chen, G. J. Snyder, Convergence of electronic bands for high performance bulk thermoelectrics. *Nature* **473**, 66–69 (2011).

53. A. J. Hong, C. L. Yuan, G. Gu, J. M. Liu, Novel p-type thermoelectric materials Cu_3MCh_4 (M = V, Nb, Ta; Ch = Se, Te): High band-degeneracy. *J. Mater. Chem. A* **5**, 9785–9792 (2017).

54. J. Wen, H. Huang, X. Yu, D. Wang, K. Guo, D. Wan, J. Luo, J.-T. Zhao, Thermoelectric properties of p-Type Cu_3VSe_4 with high seebeck coefficients. *J. Alloys Compd.* **879**, 160387 (2021).

55. Y. Liu, T. Ding, X. Luo, Y. Li, J. Long, K. Wu, Tuning intermediate-band Cu_3VS_4 nanocrystals from plasmonic-like to excitonic *via* shell-coating. *Chem. Mater.* **32**, 224–233 (2020).

56. H. Arribart, B. Sapoval, O. Gorochov, N. LeNagard, Fast ion transport at room temperature in the mixed conductor Cu_3VS_4. *Solid State Commun.* **26**, 435–439 (1978).

57. H. Arribart, B. Sapoval, Theory of mixed conduction due to cationic interstitials in the *p*-type semiconductor Cu_3VS_4. *Electrochem. Acta* **24**, 751–754 (1979).

58. H. You, Y. Jia, Z. Wu, F. Wang, H. Huang, Y. Wang, Room-temperature pyro-catalytic hydrogen generation of 2D few-layer black phosphorene under cold-hot alternation. *Nat. Commun.* **9**, 2889 (2018).

59. N. D. Donahue, H. Acar, S. Wilhelm, Concepts of nanoparticle cellular uptake, intracellular trafficking, and kinetics in nanomedicine. *Adv. Drug Deliv. Rev.* **143**, 68–96 (2019).

60. M. Sousa de Almeida, E. Susnik, B. Drasler, P. Taladriz-Blanco, A. Petri-Fink, B. Rothen-Rutishauser, Understanding nanoparticle endocytosis to improve targeting strategies in nanomedicine. *Chem. Soc. Rev.* **50**, 5397–5434 (2021).

61. X. Guo, N. Yang, W. Ji, H. Zhang, X. Dong, Z. Zhou, L. Li, H.-M. Shen, S. Q. Yao, W. Huang, Mito-bomb: Targeting mitochondria for cancer therapy. *Adv. Mater.* **33**, e2007778 (2021).

62. G. Kresse, J. Hafner, Ab initio molecular dynamics for liquid metals. *Phys. Rev. B* **47**, 558–561 (1993).

63. G. Kresse, J. Furthmüller, Efficient iterative schemes for ab initio total-energy calculations using a plane-wave basis set. *Phys. Rev. B* **54**, 11169–11186 (1996).

64. J. P. Perdew, K. Burke, M. Ernzerhof, Generalized gradient approximation made simple. *Phys. Rev. Lett.* **77**, 3865–3868 (1996).

65. P. E. Blöchl, Projector augmented-wave method. *Phys. Rev. B* **50**, 17953–17979 (1994).

66. H. J. Monkhorst, J. D. Pack, Special points for Brillouin-zone integrations. *Phys. Rev. B* **13**, 5188–5192 (1976).

67. X. S. Lv, Z. H. Deng, F. X. Miao, G. X. Gu, Y. L. Sun, Q. L. Zhang, S. M. Wan, Fundamental optical and electrical properties of nano-Cu_3VS_4 thin film. *Opt. Mater.* **34**, 1451–1454 (2012).

68. Y. Kang, Q. Zhang, C. Fan, W. Hu, C. Chen, L. Zhang, F. Yu, Y. Tian, B. Xu, High pressure synthesis and thermoelectric properties of polycrystalline Bi_2Se_3. *J. Alloys Compd.* **700**, 223–227 (2017).

69. Y. Shen, C. Li, R. Huang, R. Tian, Y. Ye, L. Pan, K. Koumoto, R. Zhang, C. Wan, Y. Wang, Eco-friendly p-type Cu_2SnS_3 thermoelectric material: Crystal structure and transport properties. *Sci. Rep.* **6**, 32501 (2016).

70. X. Su, F. Fu, Y. Yan, G. Zheng, T. Liang, Q. Zhang, X. Cheng, D. Yang, H. Chi, X. Tang, Q. Zhang, C. Uher, Self-propagating high-temperature synthesis for compound thermoelectrics and new criterion for combustion processing. *Nat. Commun.* **5**, 4908 (2014).

71. Z. Fan, H. Wang, Y. Wu, X. J. Liu, Z. P. Lu, Thermoelectric high-entropy alloys with low lattice thermal conductivity. *RSC Adv.* **6**, 52164–52170 (2016).

72. S. I. Kim, K. H. Lee, H. A. Mun, H. S. Kim, S. W. Hwang, J. W. Roh, D. J. Yang, W. H. Shin, X. S. Li, Y. H. Lee, G. J. Snyder, S. W. Kim, Dense dislocation arrays embedded in grain boundaries for high-performance bulk thermoelectrics. *Science* **348**, 109–114 (2015).

73. M. K. Jana, K. Pal, U. V. Waghmare, K. Biswas, The origin of ultralow thermal conductivity in InTe: Lone-pair-induced anharmonic rattling. *Angew. Chem. Int. Ed.* **55**, 7792–7796 (2016).
